# Engineered *Escherichia coli* platforms for tyrosine-derivative production from phenylalanine using phenylalanine hydroxylase and tetrahydrobiopterin-regeneration system

**DOI:** 10.1186/s13068-023-02365-5

**Published:** 2023-07-18

**Authors:** Yasuharu Satoh, Keita Fukui, Daisuke Koma, Ning Shen, Taek Soon Lee

**Affiliations:** 1 Faculty of Engineering, Hokkaido University, Sapporo, 060-8628 Japan; 2 Graduate School of Chemical Sciences and Engineering, Hokkaido University, Sapporo, 060-8628 Japan; 3 Research Institute for Bioscience Products & Fine Chemicals, Ajinomoto Co., Inc., Kanagawa, 210-8681 Japan; 4 Osaka Research Institute of Industrial Science and Technology, Osaka, 536-8553 Japan; 5 Biological Systems and Engineering Division, Lawrence Berkeley National Laboratory, Berkeley, CA 94720 USA

**Keywords:** Phenylalanine hydroxylase, Tyrosine, Tetrahydrobiopterin, Chromosome engineering, Tyrosol

## Abstract

**Background:**

Aromatic compounds derived from tyrosine are important and diverse chemicals that have industrial and commercial applications. Although these aromatic compounds can be obtained by extraction from natural producers, their growth is slow, and their content is low. To overcome these problems, many of them have been chemically synthesized from petroleum-based feedstocks. However, because of the environmental burden and depleting availability of feedstock, microbial cell factories are attracting much attention as sustainable and environmentally friendly processes.

**Results:**

To facilitate development of microbial cell factories for producing tyrosine derivatives, we developed simple and convenient tyrosine-producing *Escherichia coli* platforms with a bacterial phenylalanine hydroxylase, which converted phenylalanine to tyrosine with tetrahydromonapterin as a cofactor, using a synthetic biology approach. By introducing a tetrahydrobiopterin-regeneration system, the tyrosine titer of the plasmid-based engineered strain was 4.63 g/L in a medium supplemented with 5.00 g/L phenylalanine with a test tube. The strains were successfully used to produce industrially attractive compounds, such as tyrosol with a yield of 1.58 g/L by installing a tyrosol-producing module consisting of genes encoding tyrosine decarboxylase and tyramine oxidase on a plasmid. Gene integration into *E. coli* chromosomes has an advantage over the use of plasmids because it increases genetic stability without antibiotic feeding to the culture media and enables more flexible pathway engineering by accepting more plasmids with artificial pathway genes. Therefore, we constructed a plasmid-free tyrosine-producing platform by integrating five modules, comprising genes encoding the phenylalanine hydroxylase and tetrahydrobiopterin-regeneration system, into the chromosome. The platform strain could produce 1.04 g/L of 3,4-dihydroxyphenylalanine, a drug medicine, by installing a gene encoding tyrosine hydroxylase and the tetrahydrobiopterin-regeneration system on a plasmid. Moreover, by installing the tyrosol-producing module, tyrosol was produced with a yield of 1.28 g/L.

**Conclusions:**

We developed novel *E. coli* platforms for producing tyrosine from phenylalanine at multi-gram-per-liter levels in test-tube cultivation. The platforms allowed development and evaluation of microbial cell factories installing various designed tyrosine-derivative biosynthetic pathways at multi-grams-per-liter levels in test tubes.

**Supplementary Information:**

The online version contains supplementary material available at 10.1186/s13068-023-02365-5.

## Background

Aromatic compounds are an important class of diverse chemicals with a wide range of industrial and commercial applications, such as nutraceuticals (vitamin E, resveratrol, hydroxytyrosol), pharmaceuticals (3,4-dihydroxyphenylalanine [DOPA], adrenalin, morphine, melatonin), fragrance ingredients (2-phenylethanol, 3-phenylpropanol), and polymers (styrene, hydroxystyrene, tyrosol) [1–6]. These compounds can be produced by various plants, algae, fungi, and bacteria from proteinogenic amino acids, with phenylalanine (Phe), tyrosine (Tyr), and tryptophan (Trp) as precursors.

Although these aromatic compounds can be obtained by extraction from producers, their growth is slow. Additionally, the content of the compounds is low. To overcome these problems, many aromatic compounds have been chemically synthesized from petroleum-based feedstocks. However, because of the environmental burden and depleting availability of feedstock, other sustainable and environmentally friendly processes are required.

Recent remarkable advances in metabolic engineering and synthetic biology have made it possible to develop fermentative processes using microbial cell factories, which utilized natural and non-natural biosynthetic pathways to produce chemicals from renewable resources [7–11]. Aromatic compounds derived from Tyr are important chemicals and various microbial cell factories that produce Tyr derivatives have been developed by already known and artificially designed biosynthetic pathways that utilize enzymes/genes from different sources. *Escherichia coli* and yeast have been extensively used as hosts [1–4]. Several bacteria were also considered. Among the hosts, *E. coli* exhibits considerable advantages in the rapid development of microbial cell factories suitable for industrial production because of its high growth rate and well-studied genome and metabolic network as well as the availability of various synthetic biology tools for engineering and established strategies for high-cell-density fermentation in inexpensive media [5].

**Fig. 1:**
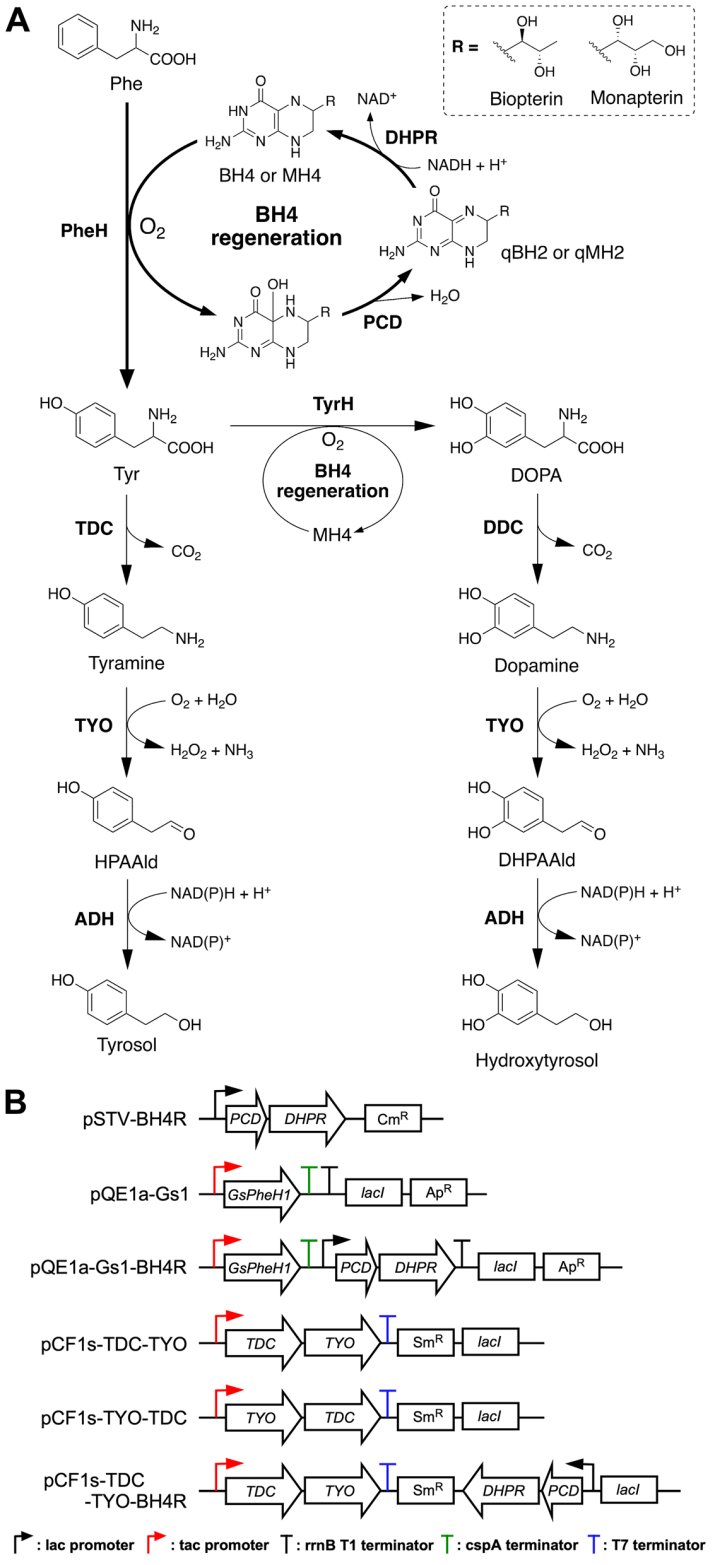
Biosynthetic pathways for producing tyrosine and its derivatives from phenylalanine (**A**) and gene organization in the constructed plasmids (**B**)

We have succeeded in engineering *E. coli* to produce DOPA, tyrosol, and hydroxytyrosol from Tyr, which was supplied via a central metabolic pathway and supplemented in cultivation media; however, the titers were low (< 1.22 mM) (Fig. 1A) [12, 13]. Therefore, for high Tyr-derivative production, enhancement of Tyr supply in *E. coli* is needed. However, Tyr production by *E. coli* is limited because its biosynthesis is elaborately regulated [14]. Furthermore, low solubility of Tyr (0.45 g/L [2.5 mM] in water at 25 °C) makes it difficult to feed Tyr into culture broths at high concentrations [15]. To increase Tyr supply, various metabolic engineering approaches, such as deregulation at transcriptional level and overexpression of bottleneck and feedback-resistant enzymes, have been employed [16–18]. Although the titers by flask-scale production were reported to be 2 to 3 g/L by the rationally engineered strains, further enhanced production is necessary for industrial applications.

Tyr can be converted from Phe by Phe hydroxylase (PheH) [19, 20]. PheH is an iron-dependent non-heme enzyme that catalyzes *para*-hydroxylation of Phe using O_2_ and tetrahydrobiopterin (BH4) as the reducing substrate (Fig. 1A). Some bacteria, including *Chromobacterium*, *Pseudomonas*, and *Xanthomonas* species, have PheHs, which use tetrahydromonapterin (MH4) instead of BH4 as the cofactor [21, 22]. Previously, we succeeded in engineering an *E. coli* strain that could oxidize Tyr to DOPA using mouse Tyr hydroxylase (TyrH), a PheH homolog, and endogenous MH4, together with the human BH4 regeneration system, which reduces the oxidized form of the cofactor, quinonoid dihydromonapterin (qMH2) [13]. In this study, we developed a simple and convenient Tyr-supplying *E. coli* strain by utilizing a bacterial PheH and the human BH4 regeneration system. The Tyr titer of the strain expressing these enzymes with a plasmid was 4.63 g/L (25.5 mM) by feeding 5.00 g/L (30.3 mM) of Phe, a highly water-soluble compound (29.6 g/L [179 mM] in water at 25 °C). To enable more flexible pathway engineering, we also constructed a plasmid-free platform, which was performed by integration of the above-mentioned genes of the PheH and the human BH4 regeneration system into the chromosome. This has an advantage over the use of plasmids because it increases genetic stability without antibiotic feeding into the culture media and accepts more plasmids carrying artificial pathway genes. These platform strains were successfully applied to produce Tyr-derived compounds, such as DOPA, tyrosol, and hydroxytyrosol.

## Results

### Screening of PheHs for Tyr-overproduction

We searched for PheHs with high activities for construction of an *E. coli* platform to produce Tyr from Phe at a high titer. First, the activity of rat PheH (RatPheH) was examined because the enzyme is well characterized and has been successfully expressed as an active form in *E. coli* [19, 23]. The RatPheH consists of *N*-terminus regulatory domain and *C*-terminus catalytic domain. A truncated enzyme lacking the regulatory domain was previously reported to have almost the same activity as the parental enzyme and to be highly expressed in *E. coli*. Therefore, a codon-optimized DNA fragment encoding only the catalytic domain of the RatPheH (RatPheHc) was synthesized and cloned into the protein expression vector pQE1a-Red (pQE1a-RatC, Table 1), in which the gene was expressed under the control of the strong *tac* promoter and repressed by *lac* operator and *lac* repressor (LacI). To estimate the net effect of PheH activity for Tyr production, a Tyr-auxotrophic mutant *E. coli* Y0 strain, in which *tyrA* encoding bifunctional chorismate mutase/prephenate dehydratase was knocked out [24], was used as host (Table 2). For regeneration of the cofactor MH4, which is stoichiometrically consumed during the Phe hydroxylation reaction, the human pterin-4α-carbinolamine dehydratase gene (*PCD*) and dihydropteridine reductase gene (*DHPR*) were also coexpressed, in that order, under the control of a *lac* promoter using plasmid pSTV-BH4R (Fig. 1B). The production of the PCD and DHPR was confirmed by Western blot analyses (Additional file 1: Fig. S1) and the strain Y0 harboring pSTV-BH4R was designated as strain YBR (Table 2).

**Fig. 2:**
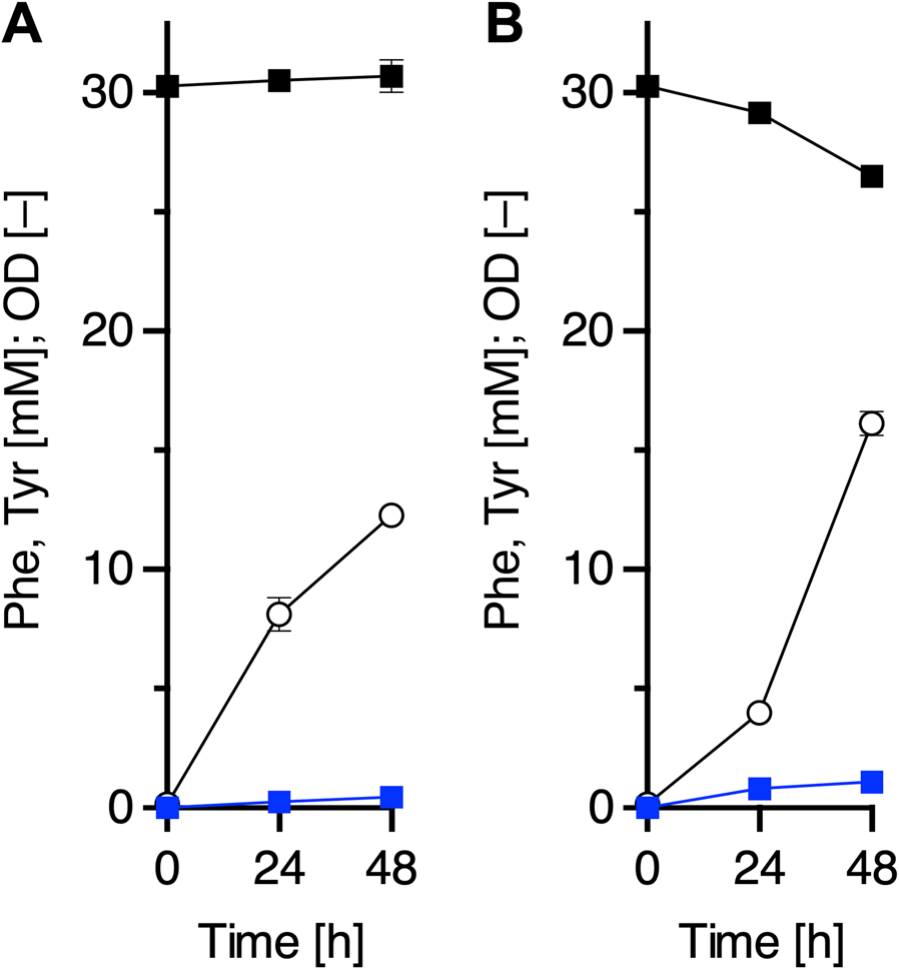
Tyrosine production of plasmid-based engineered strains with rat phenylalanine hydroxylase. Strain YBR expressing the catalytic domain of RatPheH (RatPheHc), using glucose (**A**) or glycerol (**B**) as the carbon sources. Each of the transformants was cultured up to 48 h at 30 °C. Phe, black squares; Tyr, blue squares; OD, white circles. Data are presented as mean values with standard deviations for three independent experiments. Symbols without an error bar indicate that they are larger than the size of the error bar

Then, the RatPheHc expression in strain YBR, harboring pQE1a-RatC, was analyzed by sodium dodecyl sulfate–polyacrylamide gel electrophoresis (SDS-PAGE). As shown in Additional file 1: Fig. S2, soluble expression of the RatPheHc was confirmed. To examine Tyr production, the transformant was cultured in M9Y medium, including 30.3 mM (5.00 g/L) Phe and 1.0%(w/v) glucose, for 48 h in test tubes, and the product was analyzed by high-performance liquid chromatography (HPLC). As shown in Fig. 2A, approximately 0.443 ± 0.030 mM (0.080 g/L) of Tyr was produced even though nearly all Phe remained. Therefore, we tried to improve the low conversion rate.

The oxidized form of cofactor qMH2 generated during the Phe hydroxylation reaction is reduced by DHPR with NADH. Glycerol reportedly regenerates NADH more effectively than glucose [25]; thus, glycerol was used as the sole carbon source. Although the Tyr titer was improved to 1.09 ± 0.19 mM (0.197 g/L), most of the Phe still remained in the culture medium (Fig. 2B). In addition, after 24 h of cultivation, cell growth was slower than when glucose was used as carbon source.

**Fig. 3:**
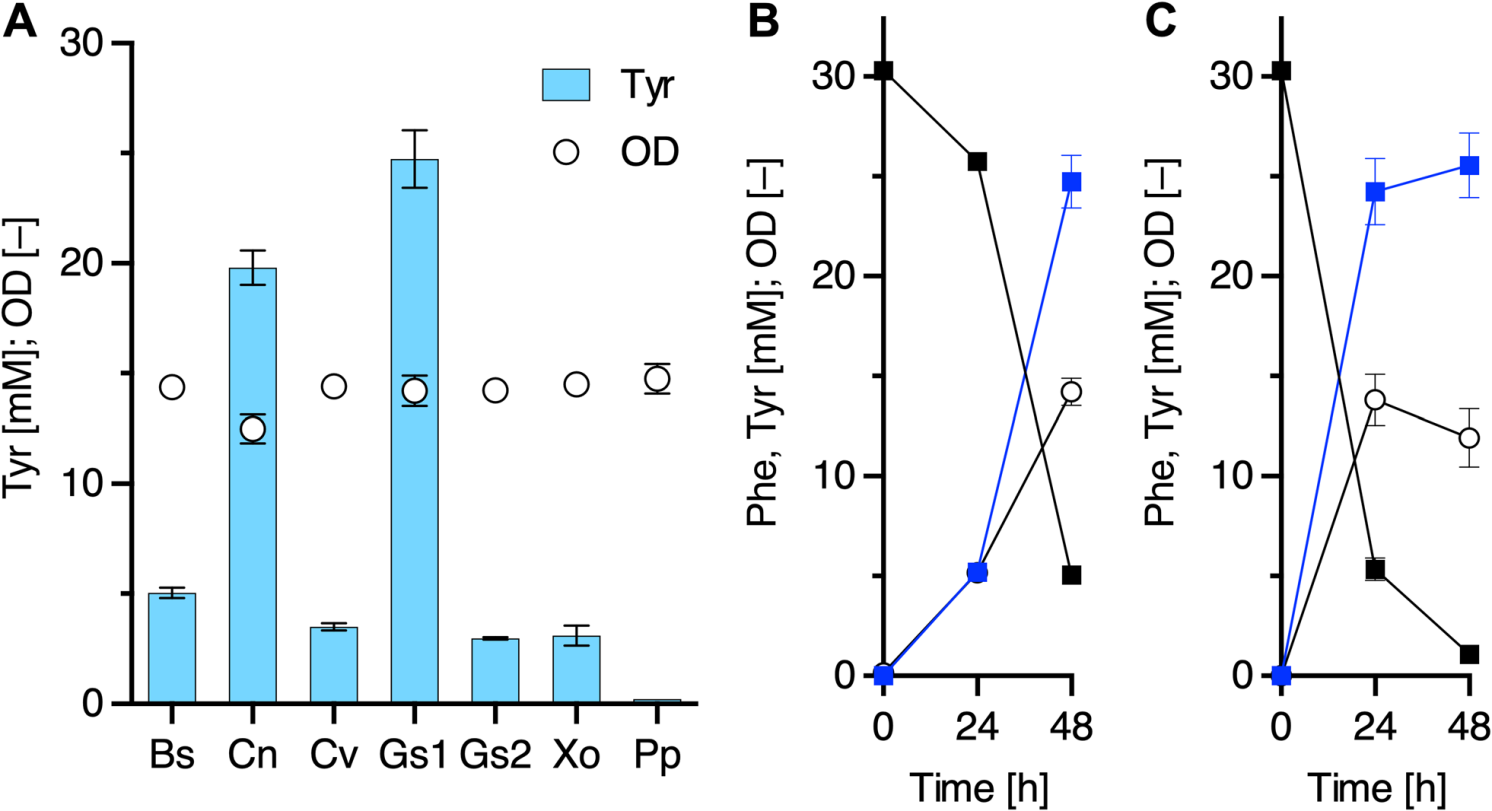
Tyrosine production of plasmid-based engineered strains with bacterial phenylalanine hydroxylases. **A** Tyrosine production of strain YBR harboring pQE1a derivatives, including bacterial PheHs. Each of the transformants was cultured for 48 h at 30 °C. Glycerol was used as the carbon source. BsPheH, Bs; CnPheH, Cn; CvPheH, Cv; GsPheH1, Gs1; GsPheH2, Gs2; XoPheH, Xo; PpPheH, Pp; OD, circles; Tyr, bars. **B** and **C** Fermentation profiles of tyrosine production of strain YBR harboring pQE1a-Gs1 (**B**) and strain PGs (**C**). Each of the transformants was cultured up to 48 h at 30 °C. Phe, black squares; Tyr, blue squares; OD, white circles. Data are presented as mean values with standard deviations for three independent experiments. Symbols without an error bar indicate that they are larger than the size of the error bar

To further improve the productivity, we next examined the activities of seven other bacterial PheHs. The genes were selected from different classes of bacteria, including *Bacillus* sp. INT005 (*BsPheH*) [26] from Bacilli; *Cupriavidus necator* (*CnPheH*), *Chromobacterium violaceum* (*CvPheH*) and *Gulbenkiania* sp. SG4523 (*GsPheH1* and *GsPheH2*) from β-proteobacteria; and *Xanthomonas oryzae* (*XoPheH*) and *Pseudomonas putida* (*PpPheH*) from γ-proteobacteria. The identities of PheHs among these enzymes are 20% to 70% (Additional file 1: Table S1). Polymerase chain reaction (PCR) amplified-DNA fragments encoding PheHs were cloned into the protein expression vector pQE1a-Red and used for Tyr production in the same manner as described above. As shown in Additional file 1: Fig. S2, we confirmed that all enzymes were produced as soluble forms in the strain YBR by SDS-PAGE analysis. In terms of Tyr production (Fig. 3A, B), a strain YBR carrying *GsPheH1* yielded the highest titer (24.7 ± 1.3 mM [4.48 g/L]) among the tested PheHs at 48 h of cultivation. Therefore, *GsPheH1* was selected for further analyses.

### Plasmid-based Tyr-producing platform

#### Construction of plasmid-based Tyr-producing platform

To construct pathways for Tyr-derivative production, many pathway genes are introduced into the host cell. From this point of view, the number of plasmids carrying genes encoding PheH and cofactor regeneration enzymes should be minimal. Therefore, we constructed plasmid pQE1a-Gs1-BH4R (Fig. 1B and Table 1), which co-expresses *GsPheH1* and the BH4-regeneration genes, which encode PCD and DHPR, as described in supplementary materials. As shown in Fig. 3C, strain Y0 harboring pQE1a-Gs1-BH4R, designated as strain PGs (Table 2), converted most of the Phe to Tyr, 25.5 ± 1.6 mM (4.63 g/L) after 48 h of cultivation. The cell growth and Tyr titer (24.2 ± 1.7 mM [4.39 g/L]) at 24 h of cultivation were markedly improved when compared with those of strain YBR harboring pQE1a-Gs1 (Fig. 3B). Therefore, we then applied strain PGs for Tyr-derivative production.

**Table 1:** Plasmids used in this study

Plasmids	Description	Sources
pSTV28	Cloning and protein expression vector; *lac* promoter, p15A ori, Cm^R^	Takara Bio
pSTV28N	pSTV28N derivative; *Nde*I site created downstream of the ribosome binding site	This study
pSTV-BH4R	pSTV28N derivative; expression of *PCD* and *DHPR* genes as an operon	This study
pQE-80L	Protein expression vector; T5 promoter, *lacI*, ColE1 ori of pBR322, Ap^R^	Qiagen
pQE1a-Red	pQE-80 derivative; protein expression vector; *tac* promoter, *lacI*, DsRed-monomer gene, ColE1 ori, Ap^R^	This study
pQE1a	pQE1a-Red derivative; deletion of DsRed-monomer gene as a control plasmid	This study
pQE1a-RatC	pQE1a-Red derivative; expression of *RatPheHc*, codon-optimized for *E. coli*	This study
pQE1a-Bs	pQE1a-Red derivative; expression of *BsPheH* gene of *Bacillus* sp.	This study
pQE1a-Cn	pQE1a-Red derivative; expression of *CnPheH* gene of *C. necator*	This study
pQE1a-Cv	pQE1a-Red derivative; expression of *CvPheH* gene of *C. violaceum*, codon-optimized for *E. coli*	This study
pQE1a-Gs1	pQE1a-Red derivative; expression of *GsPheH1* gene of *Gulbenkiania* sp.	This study
pQE1a-Gs2	pQE1a-Red derivative; expression of *GsPheH2* gene of *Gulbenkiania* sp.	This study
pQE1a-Pp	pQE1a-Red derivative; expression of *PpPheH* gene of* P. putida*	This study
pQE1a-Xo	pQE1a-Red derivative; expression of *XoPheH* gene of *X. oryzae*	This study
pQE1a-Gs1-BH4R	pQE1a-Gs1 derivative; coexpression of *GsPheH1* gene and *PCD*–*DHPR* operon	This study
pCDF1b	Protein expression vector; T7 promoter, *lacI*, CloDF13 ori, Sm^R^	Merck
pCF1s-Red	pCDF derivative; protein expression vector; *tac* promoter, *lacI*, DsRed-monomer gene	This study
pCF1s-TDC-TYO	pCF1s derivative; expression of *TDC* and *TYO* genes as an operon in this order	This study
pCF1s-TDC-TYO-BH4R	pCF1s-TDC-TYO derivative; expression of *PCD*–*DHPR* operon	This study
pCF1s-TYO-TDC	pCF1s derivative; expression of *TYO* and *TDC* genes as an operon in this order	This study
pBbS1a-2	Expression of *TYO* and *TDC* genes as an operon, *trc* promoter, *lacI*, SC101 ori, Ap^R^	[12]
pBbS1a-3	Expression of *DDC* and *TYO* genes as an operon, *trc* promoter, *lacI*, SC101 ori, Ap^R^	[13]
pBbE1k-3	Expression of *TyrH*, *DHPR*, and *PCD* genes as an operon, *trc* promoter, ColE1 ori, Km^R^	[13]

**Table 2:** Strains used in this study

Strains	Description	Sources
*Bacillus* sp. INT005	FERM P-18327, a source of *BsPheH* gene	NITE, [26]
*Cupriavidus necator* H16	ATCC 17699, a source of *CnPheH* gene	ATCC
*Gulbenkiania* sp. SG4523	NBRC 113456, a source of *GsPheH1* and *GsPheH2* genes	NITE
*Pseudomonas putida* KT2440	NBRC 100650, a source of *PpPheH* gene	NITE
*Xanthomonas oryzae*	MAFF 311018, a source of *XoPheH* gene	NARO
*Escherichia coli* JM109	*endA1*, *gyrA96*, *thi*, *hsdR17*, *supE44*, *relA1*, Δ(*lac-proAB*), *recA1*, F’[*traD36*, *proAB*^+^, *lacI*^q^, *lacZ*ΔM15]	Nippon Gene
*Escherichia coli* BW25113	Wild type; *rrnB3* ∆*lacZ4787 hsdR514* ∆(*araBAD*)*567* ∆(*rhaBAD*)*568 rph-1*	NIG
Y0Km	BW25113 derivative, ∆*tyrA* with Km^R^-marker, ∆*feaB*-*tynA* without Km^R^-marker	This study
Y0	Y0Km derivative, ∆*tyrA* without Km^R^-marker	This study
YBR	Y0 harboring pSTV-BH4R	This study
PGs	Y0 harboring pQE1a-Gs1-BH4R	This study
GsBR1	Y0Km derivative, Tyr-producing gene cassette integrated at *tyrA*-knockout region	This study
GsBR2	GsBR1 derivative, Tyr-producing gene cassette integrated at *feaB*–*tynA*-knockout region	This study
GsBR2∆*aroD*	GsBR2 derivative, ∆*aroD* with Km^R^-marker	This study
GsBR3	GsBR2∆*aroD* derivative, Tyr-producing gene cassette integrated downstream of *aroD* locus	This study
GsBR3∆*cysE*	GsBR3 derivative, ∆*cysE* with Km^R^-marker	This study
GsBR4	GsBR3∆*cysE* derivative, Tyr-producing gene cassette integrated downstream of *cysE* locus	This study
GsBR4∆*serA*	GsBR4 derivative, ∆*serA* with Km^R^-marker	This study
GsBR5	GsBR4∆*serA* derivative, Tyr-producing gene cassette integrated downstream of *serA* locus	This study

NITE, National Institute of Technology and Evaluation, Tokyo, Japan

ATCC, American Type Culture Collection, Manassas, VA, USA

NARO, National Agriculture and Food Research Organization, Ibaraki, Japan

Nippon Gene Co., LTD., Tokyo, Japan

NIG, National Institute of Genetics, Shizuoka, Japan

#### Application of a plasmid-based Tyr-producing platform strain

**Fig. 4:**
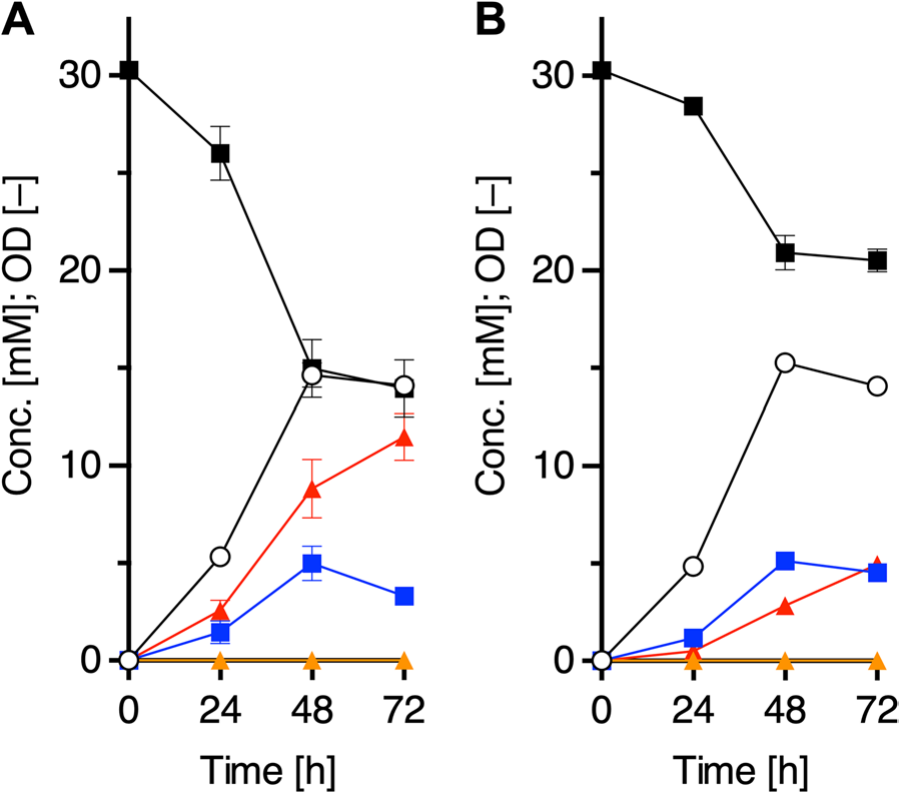
Tyrosol production of plasmid-based engineered strains. Fermentation profiles of tyrosol production of strain PGs harboring pCF1s-TDC-TYO (**A**) or pCF1s-TYO-TDC (**B**). Each of the transformants was cultured up to 72 h at 30 °C. Phe, black squares; Tyr, blue squares; tyramine, orange triangles; tyrosol, red triangles; OD, white circles. Data are presented as mean values with standard deviations for three independent experiments. Symbols without an error bar indicate that they are larger than the size of the error bar

We evaluated the above-mentioned plasmid-based Tyr-producing platform by measuring tyrosol productivity. Tyrosol is an attractive phenolic compound used for pharmaceuticals and fine chemicals [27–29]. We have constructed a tyrosol biosynthetic pathway from Tyr via three steps: decarboxylation of Tyr to tyramine, deamination of tyramine to 4-hydroxyphenylacetaldehyde (HPAAld), and reduction of HPAAld to tyrosol (Fig. 1A) [12]. As endogenous enzyme(s) in *E. coli*, such as alcohol dehydrogenase(s), can catalyze the reduction of HPAAld to tyrosol, two genes encoding Tyr decarboxylase (TDC) from *Papaver somniferum* and tyramine oxidase (TYO) from *Micrococcus luteus* were introduced into the Tyr producer. We previously constructed a plasmid, pBbS1a-2, which carried the TDC- and TYO-encoding genes, but the selection marker was ampicillin-resistance (Ap^R^), which is the same as pQE1a-Gs1-BH4R. We therefore reconstructed plasmids with a pCF1s-Red vector (streptomycin-resistance marker [Sm^R^], pCDF ori) as described in supplementary materials. The TDC- and TYO-encoding genes were cloned as artificial operons into pCF1s-Red, so that the order of the two genes was interchanged to make pCF1s-TDC-TYO and pCF1s-TYO-TDC, respectively (Fig. 1B). When the transformants were cultured in M9Y medium with 30.3 mM (5.00 g/L) Phe for 72 h at 30 ºC, tyrosol was produced with a yield of 4.93 ± 0.31 mM (0.682 g/L) by strain PGs harboring pCF1s-TYO-TDC, while 11.5 ± 1.2 mM (1.58 g/L) was yielded by strain PG harboring pCF1s-TDC-TYO, which is 2.3-fold higher than that of the former strain (Fig. 4A, B). The results suggested that the gene order in the operon was crucial for increased titer. Thus, we demonstrated that the platform could be applicable for Tyr-derivative-producing pathways.

### Plasmid-free Tyr-producing platform

#### Integration of Tyr-producing module into *E. coli* chromosome

Gene integration into the *E. coli* chromosome offers considerable advantages over the use of plasmids, especially for large-scale industrial applications [18, 30]. It increases genetic stability without antibiotic feeding to the culture media and enables more flexible pathway engineering as more plasmids carrying artificial pathway genes are acceptable. Therefore, we attempted to develop a plasmid-free Tyr-supplying platform *E. coli* strain by integrating *GsPheH1* and BH4-regeneration-related genes into the chromosome as a cassette (Tyr-producing module).

**Fig. 5:**
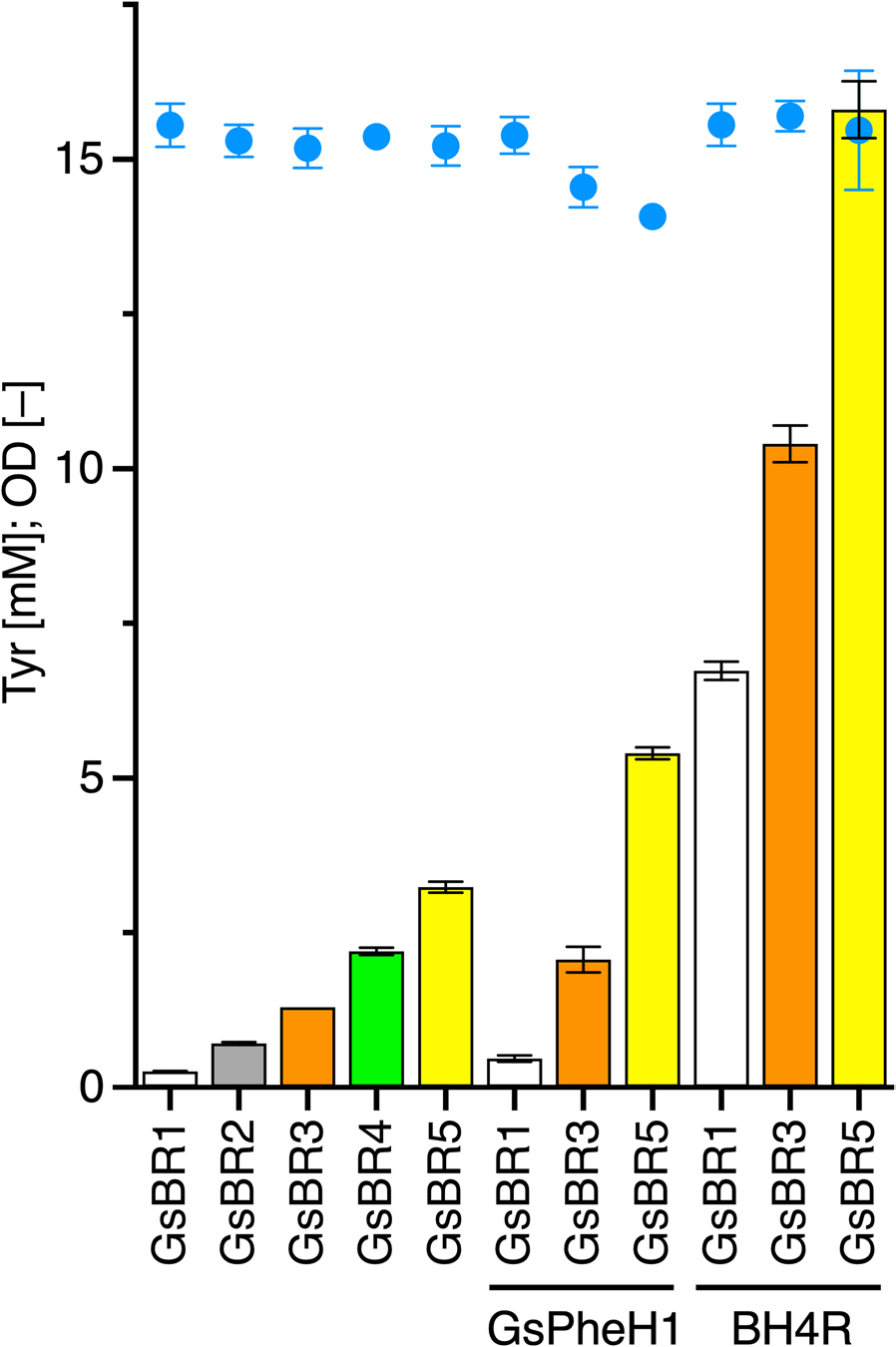
Tyrosine production of chromosome engineered strains GsBR1 to GsBR5. Strains GsBR1 (white), GsBR2 (gray), GsBR3 (orange), GsBR4 (green), and GsBR5 (yellow), in which one to five Tyr-producing modules were integrated at different gene loci on the chromosome, were tested. Strains GsBR1, GsBR3, and GsBR5 transformed with pQE1a-Gs1 (GsPheH1) or pSTV-BH4R (BH4R) were also evaluated. Each strain was cultured for 48 h at 30 °C. Tyr, bars; OD, blue circles. Data are presented as mean values with standard deviations for three independent experiments. Symbols without an error bar indicate that they are larger than the size of the error bar

For integration of the Tyr-producing module into the *E. coli* chromosome, we employed the bacteriophage λRed recombineering system [31–33]. As an integration site, we selected the *tyrA* locus because the *tyrA*-knockout mutant Y0 could be recovered by introduction of *GsPheH1* and BH4-regeneration-related genes as described above. As described in supplementary materials, the desired strain GsBR1 was successfully obtained and then evaluated for its Tyr production (Fig. 5). When cultivated under the same conditions mentioned above, Tyr productivity was markedly decreased, to 0.252 ± 0.012 mM (0.046 g/L), when compared with that of the plasmid-based strain PGs (Fig. 3C). Considering that the copy number of pQE vectors used for the plasmid-based platform is 20 to 30 (Qiagen, Dusseldorf, Germany), the low productivity was likely due to gene dosage of the Tyr-producing module.

To improve Tyr productivity, the module was additionally integrated into the *feaB*–*tynA* region of strain GsBR1 because the region was already knocked out. We obtained the strain by the method described in the supplementary materials and it was designated as strain GsBR2. The Tyr titer of the constructed strain was slightly enhanced to 0.705 ± 0.023 mM (0.128 g/L), compared to that of strain GsBR1 (Fig. 5), but was still lower than that of the plasmid-based strain PGs.

#### Stepwise and scarless integration of the Tyr-supplying module at different locations of the chromosome using λRed recombinase

**Fig. 6:**
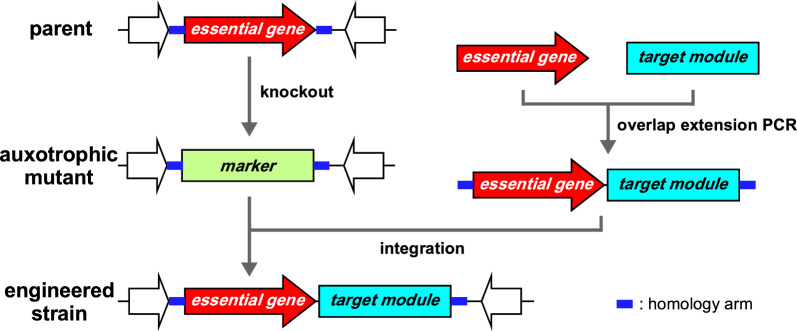
Schematic diagram of scarless chromosome engineering using λRed recombinase. First, an auxotrophic mutant with an essential gene knocked out is constructed using λRed recombinase and an antibiotic-resistance marker. The auxotrophic phenotype of the mutant is then recovered using λRed recombinase and a DNA fragment in which the essential gene and a target module are assembled by overlap extension polymerase chain reaction

As demonstrated with the construction of the strains, GsBR1 and GsBR2, λRed recombineering is a powerful tool for integration of a DNA fragment prepared by PCR into the desired chromosomal site of *E. coli*. In general, an antibiotic-resistance marker is repeatedly used for gene integration and knockout and is removed with flippase (FLP)/FLP recognition target (FRT) recombination for marker recycling [32, 33]. However, multiple FRT-sequences (scars) left on the chromosome can induce chromosomal deletion and rearrangements between undesired FRT-sequences in the FLP/FRT recombination reaction. We therefore attempted to develop a genome-engineering method without scar sequences based on λRed-based recombineering and auxotrophy complementation. The scheme depicting our process is shown in Fig. 6. At first, an essential gene for *E. coli* was selected as the target locus for module integration and its knockout mutant was constructed by the λRed-mediated recombination method, using an appropriate antibiotic-resistance gene. The auxotrophic mutant was then transformed with λRed recombinase and the DNA fragment assembled the essential gene, the target module, and attached homology arms for chromosome integration. Finally, DNA integration in recombinant cells showing recovery of the auxotrophic phenotype was confirmed by PCR and sequence analysis. To reduce unexpected effects on downstream genes by the module integration, we selected essential genes that were least likely to form an operon structure with downstream genes.

To examine the effectivity of this strategy, *aroD* (3-dehydroquinate dehydratase gene), essential for aromatic amino acid production [24, 34], was targeted. A DNA fragment, comprised *aroD* and the Tyr-producing module, was replaced with the kanamycin (Km)-resistance marker in the chromosome of an *aroD*-knockout mutant which was derived from strain GsBR2 (strain GsBR2∆*aroD*) as described in supplementary materials. After selection in the M9 minimal medium, we successfully obtained a recombinant strain (GsBR3). Furthermore, the Tyr-producing module was also integrated downstream of *cysE* (serine *O*-acetyltransferase gene) and *serA* (3-phosphoglycerate dehydrogenase gene)*,* in the same manner as for *aroD*, to construct strains GsBR4 and GsBR5, respectively (supplementary materials). As shown in Fig. 5, depending on the number of the Tyr-producing modules, Tyr production by strains GsBR3 to GsBR5 after 48 h of cultivation was almost linearly enhanced up to 3.23 ± 0.09 mM (0.586 g/L), which was approximately 13-fold higher than that of strain GsBR1. However, the titer of strain GsBR5 was merely 12.6% of that of strain PGs.

#### Application of plasmid-free Tyr-producing platform for its derivatives production

**Fig. 7:**
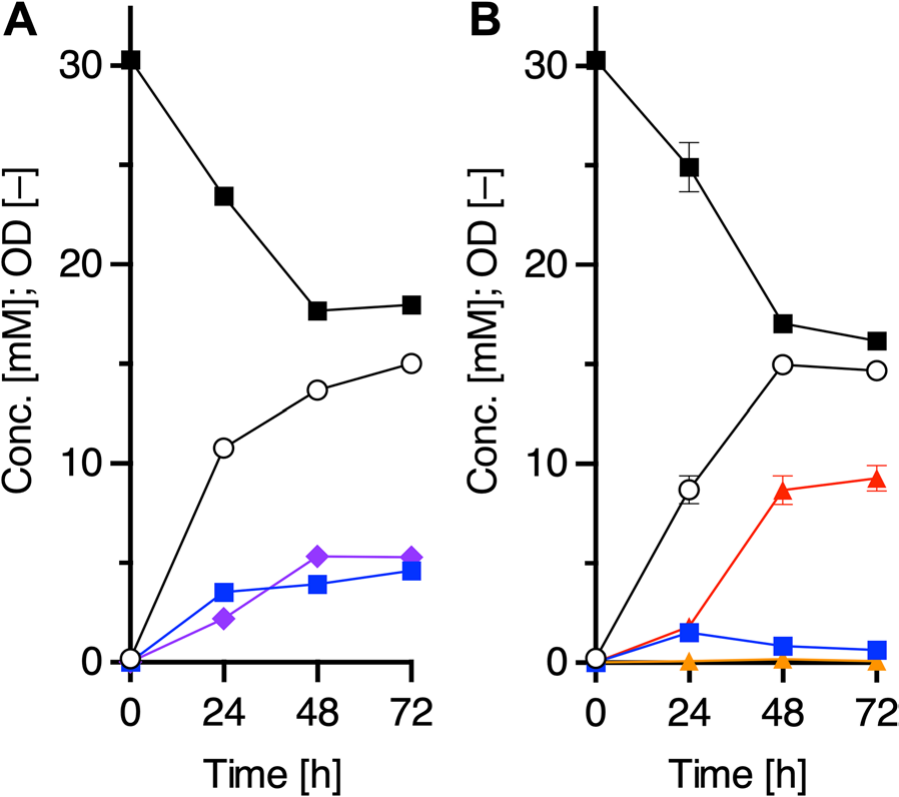
DOPA and tyrosol production of chromosome engineered strain GsBR5. DOPA (**A**) and tyrosol (**B**) production of recombinant GsBR5. Strain GsBR5 was transformed with pBbE1k-3 for DOPA production and pCF1s-TDC-TYO-BH4R for tyrosol production, respectively. Each strain was cultured up to 72 h at 30 °C. Phe, black squares; Tyr, blue squares; DOPA, purple diamonds; tyramine, orange triangles; tyrosol, red triangles; OD, white circles. Data are presented as mean values with standard deviations for three independent experiments. Symbols without an error bar indicate that they are larger than the size of the error bar

We next evaluated the plasmid-free platform based on DOPA and tyrosol production. We first examined the production of DOPA, which is used as a drug for treatment of Parkinson’s disease [35]. We previously reported that DOPA was produced from Tyr in *E. coli* expressing the mouse TyrH-encoding gene together with human BH4-regeneration-related genes on the pBbE1k-3 plasmid [13]. We therefore used this plasmid, which included *TyrH*, *DHPR*, and *PCD* as an operon in this order, under the control of *trc* promoter (DOPA-producing module). The DOPA productivity of transformant of strain GsBR5 harboring pBbE1k-3 (Fig. 7A) was 5.28 ± 0.04 mM (1.04 g/L), demonstrating that strain GsBR5 can be used to produce DOPA. Interestingly, the titer exceeded Tyr production (3.23 mM) of the host strain GsBR5, suggesting that the BH4-regeneration system, additionally introduced by the plasmid pBbE1k-3, elevated Tyr production. To investigate this speculation, Tyr production of strain GsBR5 harboring pSTV-BH4R was examined (Fig. 5). As expected, the titer was markedly increased and reached 15.8 ± 0.5 mM (2.86 g/L) after 48 h of cultivation. In contrast, additional GsPheH1 expression was poorly effective for Tyr production (Fig. 5). Taken together, the cofactor regeneration step is a bottleneck in strain GsRB5.

Next, we evaluated tyrosol production using pCF1s-TDC-TYO. This transformant of strain GsBR5 produced 4.41 ± 0.20 mM (0.609 g/L) tyrosol at 72 h. Furthermore, strain GsBR5 harboring pCF1s-TDC-TYO-BH4R, a derivative of pCF1s-TDC-TYO that had the BH4-regeneration genes inserted to improve the rate-limiting step of the host strain, produced 2.1-fold more tyrosol (9.27 ± 0.64 mM [1.28 g/L]) (Fig. 7B), which was comparable to that of the plasmid-based platform with strain PGs harboring pCF1s-TDC-TYO (11.5 mM), indicating that the additional introduction of the BH4-regeneration system was effective for increased production.

**Fig. 8:**
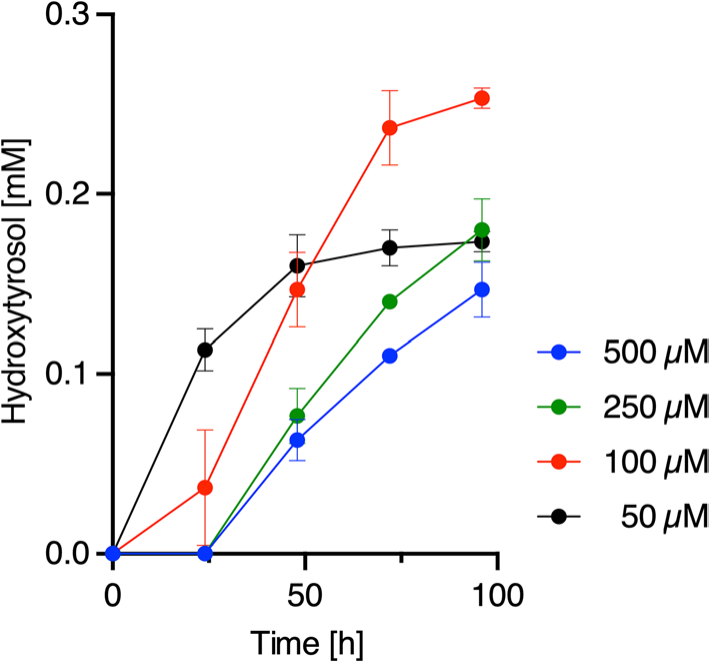
Hydroxytyrosol production of chromosome engineered strain GsBR5. Hydroxytyrosol production of recombinant GsBR5 transformed with pBbE1k-3 and pBbS1a-3. The recombinant cell, treated at different IPTG concentration to induce target protein production, was cultured up to 96 h at 30 °C. Data are presented as mean values with standard deviations for three independent experiments. Symbols without an error bar indicate that they are larger than the size of the error bar

Furthermore, we attempted to convert strain GsBR5 harboring the DOPA-producing module to hydroxytyrosol-producing cells. Hydroxytyrosol is a powerful antioxidant and used for human health promotion [36, 37]. Hydroxytyrosol is obtained from DOPA by three steps similar to those involved in tyrosol production; decarboxylation of DOPA, deamination of dopamine, and reduction of 3,4-dihydroxyphenylacetaldehyde (DHPAAld, Fig. 1A) [13]. For specific production of hydroxytyrosol without byproducts, a DOPA-specific decarboxylase (DDC) from *Sus scrofa,* which does not recognize Tyr [38], was used. Since hydroxytyrosol is obtained from dopamine by TYO from *M. luteus,* and endogenous alcohol dehydrogenase(s) in *E. coli*, the already-constructed plasmid pBbS1a-3, which includes the genes encoding DDC and TYO as an operon in this order under the control of a *trc* promoter (hydroxytyrosol-producing module), was used. Strain GsBR5 harboring pBbE1k-3 and pBbS1a-3 was cultured using the same procedures described above. As shown in Fig. 8, 0.147 ± 0.015 mM (0.023 g/L) hydroxytyrosol was produced. This titer was rather low, considering that DOPA production of strain GsBR5 harboring pBbE1k-3 was 5.28 (1.04 g/L, Fig. 7A). Since growth inhibition of GsBR5 harboring pBbE1k-3 and pBbS1a-3 was observed (Additional file 1: Fig. S3), additional expression of *DDC* and *TYO* would negatively affect the cell growth. Therefore, we cultivated the strain under various conditions by varying isopropyl-β-d-thiogalactopyranoside (IPTG) concentrations. Consequently, growth inhibition was relieved depending on a decrease in IPTG concentration (Additional file 1: Fig. S3). The titer of hydroxytyrosol production was also enhanced to 0.253 ± 0.006 mM (0.039 g/L) by addition of 100 µM IPTG (Fig. 8). Thus, strain GsBR5 could be applied to develop microbial cell factories with multi-modules for Tyr-derivative production.

## Discussion

In this study, we reported novel *E. coli* platforms for producing Tyr from Phe, using *Gulbenkiania* sp. PheH1 together with human BH4-regeneration system, at multi-gram-per-liter levels in test-tube cultivation. The titer of our engineered strain using a plasmid (strain PGs) was higher than those of rationally engineered Tyr-overproducing strains in flask cultivation (3.0 g/L, 16.6 mM) [17]. In addition, our developed platform strains were successfully applied for producing industrially valuable aromatic compounds, DOPA, tyrosol, and hydroxytyrosol, and the titers were improved, compared to those previously reported [12, 13]. Furthermore, we successfully optimized the tyrosol-producing module and revealed a bottleneck step in the hydroxytyrosol-producing pathway. Therefore, the engineered strains would be useful for the efficient development of already known and artificially designed biosynthetic pathways. Moreover, this would enable us easy access to adequate amount of rare natural Tyr-derivatives for further analysis.

In terms of hydroxytyrosol production, the titer of strain GsBR5 as a host (0.253 mM, 0.039 g/L) was improved compared with that (0.19 mM, 0.029 g/L) of *E. coli* ∆*feaB* with both DOPA- and hydroxytyrosol-producing modules in cultures fed 1 mM Tyr [13]**.** However, it was quite low, considering that the strain GsBR5 with the DOPA-producing module produced 5.28 mM (1.04 g/L) of DOPA. Recently, Nakagawa et al. reported that rat TyrH activity was inhibited by DDC from *Pseudomonas putida* [39]. In our experiment, mouse TyrH activity was likely inhibited by pig DDC. We need further analysis to elucidate this inhibition effect to improve productivity.

Effective DOPA-producing pathways in microbial cells have garnered much attention for fermentative production of natural plant products, such as the benzylisoquinoline alkaloids, morphine and codeine, and the pigment betalains [39–42]. For benzylisoquinoline alkaloid production, a tyrosinase has been used to produce DOPA from Tyr. The enzyme catalyzes multiple oxidation reactions, including Tyr to DOPA and DOPA to *ortho*-quinone, using molecular oxygen. This overoxidation results in low product yield. In contrast, the yield has been improved by utilization of monooxygenase TyrH from *Drosophila melanogaster,* overcoming the overoxidation issue of tyrosinase. Therefore, TyrHs are widely applicable for development of microbial cell factories that can produce various DOPA derivatives. Since the accumulation of Tyr (4.61 mM) was detected under our experimental conditions, as shown in Fig. 7A, we need to optimize the cultivation conditions and/or search for and engineer more active enzymes for increased production of DOPA.

To facilitate engineering of microbial cell factories in a high-throughput fashion, Design–Build–Test–Learn cycles can be applied for optimization and fine-tuning of the designed biosynthetic pathways. In these cycles, combinatorial DNA parts, consisting of the relevant genes with promoters of different strength, ribosome binding sites of different translation efficiency, and artificial operons in various gene orders, are constructed, introduced into the microbial cells, and evaluated in parallel. The key cultivation parameters are monitored in real time using optical measurement systems [43]. Recent advancements in in vivo biosensors, used to evaluate the concentration of products and intermediates, coupled to fluorescence proteins, which produce a real-time output signal, make the optical measurement more sensitive and reliable. However, Tyr precipitates would interfere with these optical measurements, owing to the addition of the reactant to the medium at high concentrations. In contrast, the platform strains preventing the issue would accelerate the efficient development of microbial cell factories.

Our ultimate goal was the development of microbial cell factories to produce Tyr-derivatives from renewable resources of biomass. To achieve this, the pathways (modules), optimized by our platform strains, can be installed into strains already engineered for Tyr-overproduction from biomass. Phe-overproducing strains can also be employed by installing the pathways (modules) together with PheH and a BH4-regeneration system. In fact, Huang et al. reported production of 2.21 mM (0.401 g/L) Tyr by a strain overexpressing a bacterial PheH gene, as well as genes responsible for the shikimate pathway and MH4 biosynthesis and recycling [44]**.** The Phe producers with a PheH may have advantages over Tyr producers, because the Phe titer of engineered strains (over 6 g/L) is higher than the Tyr titer of Tyr producers [45, 46]. In addition, some Tyr producers are *pheA* knockout mutants (Phe auxotrophy) due to the increased metabolic flux toward Tyr from chorismate and require Phe supplementation [18]. Conversely, Phe producers with PheH do not require Phe supplementation. As another approach, we would employ modular co-culture metabolic engineering approach [47–49]. In this case, the strains with Tyr-derivative-producing module(s) produce appropriate compounds using Phe, which is biosynthesized from biomass by the Phe producer, under co-culture conditions.

Remarkable progress of Clustered Regularly Interspaced Short Palindromic Repeats (CRISPR)/CRISPR-associated protein (Cas) technology in recent years has vastly facilitated genome editing of various organisms, including prokaryotes and eukaryotes. This technology is also now used widely for chromosome engineering to develop metabolic engineered strains [11, 50]. Compared to the described method that uses λRed recombinase, CRISPR/Cas technology allows for more flexible scarless integration of a DNA fragment into the chromosome. However, this system poses the risk of off-target effects, which induces mutation at untargeted sites. To reduce this unwanted effect, selection of appropriate CRISPR/Cas tools and careful design of a guide-RNA sequence are needed. Taking this into account, the scarless gene integration method based on the commonly used λRed recombination would be advantageous.

## Conclusions

In this study, we developed simple and convenient Tyr-producing *E. coli* platforms, which employ a bacterial PheH and a human BH4-regeneration system, using endogenous MH4 as a cofactor. These platforms produced Tyr in multi-gram-per-liter levels from Phe supplemented as the substrate. These platforms allowed development and evaluation of various designed Tyr-derivative biosynthetic pathways. The usefulness of the platforms was demonstrated using DOPA, tyrosol, and hydroxytyrosol production as examples. Furthermore, to facilitate development of chromosome engineering strains for metabolic engineering, we showed a scarless gene integration method based on the well-established λRed recombineering system combined with complementation of auxotrophic phenotypes.

## Materials and methods

### General

All media, chemicals, and reagents were of analytical grade and were purchased from FUJIFILM Wako Pure Chemical Corporation (Osaka, Japan), Sigma-Aldrich Japan K.K. (Tokyo, Japan), KANTO CHEMICAL Co., Inc. (Tokyo, Japan), or Tokyo Chemical Industry Co., Ltd. (Tokyo, Japan). Synthetic genes were purchased from Integrated DNA Technologies, Inc. (Coralville, IA, USA). PCR was performed using a GeneAmp PCR System 9700 thermal cycler (Thermo Fisher Scientific Inc., Waltham, MA, USA) with KOD DNA polymerase (Toyobo Co. Ltd, Osaka, Japan) according to the manufacturer’s protocols. General genetic manipulations of *E. coli* were performed according to standard protocols.

### Bacterial strains and cultures

The strains used in this study are summarized in Table 2. *Escherichia coli* JM109 (Nippon Gene Co., Ltd, Tokyo, Japan) was routinely used for plasmid construction. For Tyr production, *E. coli* BW25113 derivatives were used.

The growth medium routinely used was LB broth medium (Lennox; Sigma-Aldrich Japan K.K.). M9 minimal medium [M9 minimal salts (Becton, Dickinson and Company, Franklin Lakes, NJ, USA), 0.4 or 1.0%(w/v) carbon sources (glucose or glycerol), 5 mM MgSO_4_, 0.1 mM CaCl_2_] supplemented with 0.1%(w/v) yeast extract (M9Y medium) was used for Tyr production. Ampicillin (Ap), chloramphenicol (Cm), streptomycin (Sm), and kanamycin (Km) were added to media at 100, 30, 20, and 25 mg/L, respectively, to maintain plasmids. For the selection of gene knockout mutants, Km was used at 13 mg/L.

### Plasmid construction

Plasmids used in this study are listed in Table 1 and Fig. 1B. Detailed methods for plasmid construction are described in the supplementary materials.

### Production of Tyr and its derivatives

*Escherichia coli* strains harboring appropriate plasmids were pre-cultured in M9Y medium containing 0.4%(w/v) glucose or glycerol for 16 h at 30 °C. After inoculating appropriate amounts of the precultures into 3 mL of M9Y medium so that optical density (OD) at 600 nm to 0.15, they were incubated at 30 °C with shaking (200 rpm). The medium contained 5.00 g/L (30.3 mM) Phe, 20 mg/L FeSO_4_·7H_2_O, and 10 g/L (1.0%[w/v]) of the same carbon sources used for pre-cultivation with test tubes. To induce protein expression, IPTG was added to a final concentration of 500 µM at 4 h of cultivation, unless noted otherwise. Samples (300 µL) were collected at appropriate time-points and were analyzed by HPLC. OD measurements at 600 nm were also taken using a NanoDrop 2000C spectrophotometer (Thermo Fisher Scientific Inc.), using cuvettes after dilution in a 1 N HCl solution.

### HPLC analysis

Culture aliquots (50 µL) mixed with 1 N HCl (200 µL) were heated at 50 °C for 30 min. After centrifugation, the supernatants (2 μL) were analyzed using a Shimadzu HPLC system (Shimadzu Co., Kyoto, Japan), equipped with an InertSustain C18 column (column length, 150 mm; inner diameter, 2.1 mm; particle size, 3 μm; GL Science Inc., Tokyo, Japan). Buffer A (0.1%[v/v] formic acid solution) and buffer B (methanol with 0.1%[v/v] formic acid) were used as a mobile phase, and compounds were eluted at 35 °C and a flow rate of 0.2 mL/min, with increasing concentrations of buffer B as follows: 2%, 0 − 3 min; 2–30%, 3–35 min. Eluted compounds were detected by measuring absorbance at 210 and 280 nm.

## Supplementary Information

**Additional file 1: Figure S1.** SDS-PAGE and Western bolt analyses of PCD and DHPR production. **Figure S2.** SDS-PAGE analysis of PheH production. **Figure S3.** Fermentation profiles of strain GsBR5 transformed with pBbE1k-3 and pBbS1a-3. **Table S1.** Identities of amino acid sequences of PheHs. **Table S2.** Primers used in this study.

## Data Availability

All data generated or analyzed during this study are included in this published article and its supplementary information files.

## Abbreviations

Ap: Ampicillin
BH4: Tetrahydrobiopterin
Cas: CRISPR-associated protein
Cm: Chloramphenicol
CRISPR: Clustered regularly interspaced short palindromic repeats
DHPR: Dihydropteridine reductase
DOPA: 3,4-Dihydroxyphenylalanine
FLP: Flippase
FRT: Flippase recognition target
HPAAld: 4-Hydroxyphenylacetaldehyde
HPLC: High-performance liquid chromatography
IPTG: Isopropyl-β-d-thiogalactopyranoside
Km: Kanamycin
MH4: Tetrahydromonapterin
OD: Optical density
PCD: Pterin-4α-carbinolamine dehydratase
Phe: Phenylalanine
PheH: Phenylalanine hydroxylase
qMH2: Quinonoid dihydromonapterin
RatPheH: Rat phenylalanine hydroxylase
RatPheHc: Catalytic domain of rat phenylalanine hydroxylase
Sm: Streptomycin
TDC: Tyrosine decarboxylase
Trp: Tryptophan
TYO: Tyramine oxidase
Tyr: Tyrosine
TyrH: Tyrosine hydroxylase
