# Supplementary material for: Engineered Escherichia coli platforms for tyrosine-derivative production from phenylalanine using phenylalanine hydroxylase and tetrahydrobiopterin-regeneration system
Source: Biotechnol Biofuels Bioprod. 2023 Jul 18;16:115. doi: 10.1186/s13068-023-02365-5 (PMC10354952; doi:10.1186/s13068-023-02365-5)
Supplement: Supplementary file 1 — Additional file 1: Figure S1. SDS-PAGE and Western bolt analyses of PCD and DHPR production. Figure S2. SDS-PAGE analysis of PheH production. Figure S3. Fermentation profiles of strain GsBR5 transformed with pBbE1k-3 and pBbS1a-3. Table S1. Identities of amino acid sequences of PheHs. Table S2. Primers used in this study. [file 13068_2023_2365_MOESM1_ESM.pdf]

Additional Information

Engineered *Escherichia coli* platforms for tyrosine-derivative production from phenylalanine using phenylalanine hydroxylase and tetrahydrobiopterin-regeneration system

Yasuharu Satoh*, Keita Fukui, Daisuke Koma, Ning Shen, Taek Soon Lee

1. SDS-PAGE and Western blot analyses

Proteins were separated by SDS-PAGE and visualized with Coomassie Brilliant Blue. For Western blot analysis, proteins separated by SDS-PAGE were transferred to a polyvinylidene difluoride (PVDF) membrane using a semi-dry blotting system (ATTO Co., Tokyo, Japan). PCD and DHPR were detected using rabbit anti-human PCD and DHPR antibodies (Sigma-Aldrich Japan K.K., Tokyo, Japan) as primary antibodies, respectively, and a horseradish peroxidase-conjugated goat anti-rabbit IgG (Sigma-Aldrich Japan) as a secondary antibody. Proteins that cross-reacted with the antibodies were visualized using Western BLoT Quant HRP substrate (Takara Bio Inc., Shiga, Japan), and data were obtained with ChemiDoc XRS system (Bio-Rad Laboratories, Inc., Hercules, CA, USA).

2. Plasmid construction

Construction of protein expression vector pQE1a-Red

Protein expression vector pQE1a-Red, which included *tac* promoter, *lac* operator, a ribosome binding site, DsRed monomer (a red fluorescent protein) gene, *lac* repressor gene (*lacI*), ColE1 ori, and β-lactamase gene (for ampicillin resistance; Ap^R), and which was compatible with the BglBricks standard **[1]**, was constructed using pQE-80L vector (Qiagen K.K., Tokyo, Japan).

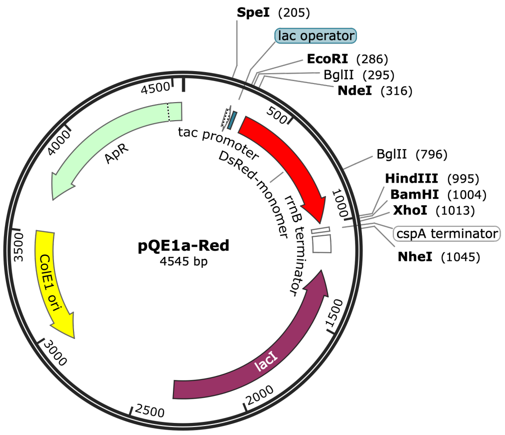

The plasmid backbone was prepared from pQE-80L vector as follows: to delete *Nde*I site in the mob region of pQE-80L, the vector digested with *Nde*I was blunted and self-ligated using Blunting high kit (Toyobo Co., Ltd, Osaka, Japan). The resulting constructed vector was used as a template for PCR with primers QE1 and QE2 (Table S2) to obtain the plasmid backbone, except for T5 promoter and chloramphenicol acetyltransferase gene.

The DsRed gene, controlled by *tac* promoter and *lac* operator, was prepared by 2-steps PCR. The first PCR step was carried out using pDsRed-Monomer (Takara Bio Inc.) as a template and QE3 and QE4 as primers (Table S2). The second PCR was then performed using the amplicon as the template and QE5 and QE6 as primers (Table S2). Finally, the amplicons,

including the plasmid backbone and DsRed gene, were treated with *Sal*I and *Nhe*I and ligated to obtain pQE1a-Red vector.

Construction of protein expression vector pCF1s-Red

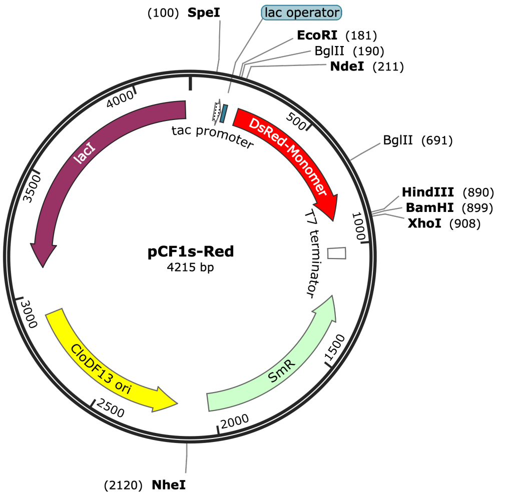

Protein expression vector pCF1s-Red, which included *tac* promoter, *lac* operator, a ribosome binding site, DsRed monomer gene, *lac* repressor gene (*lacI*), CloDF13 ori, and streptomycin resistance marker gene (Sm^R), and which was compatible to the BglBricks standard **[1]**, was constructed based on pCDF-1b vector (Merck KGaA, Darmstadt, Germany).

Plasmid backbone was prepared by PCR using the pCDF-1b vector as a template and CF1 and CF2 as primers (Table S2). The amplicon and DsRed-Monomer gene in pQE1a-Red, which was digested with *Spe*I and *Xho*I, were ligated to obtain pCF1s-Red vector.

Construction of protein expression vector pSTV28N

Protein expression vector pSTV28N, which included *lac* promoter, *lac* operator, a ribosome binding site, p15A ori, and chloramphenicol resistance marker gene (Cm^R), was constructed using pSTV28 vector (Takara Bio Inc.). For effective protein expression, *Nde*I site was also created downstream of the ribosome binding site, using PCR.

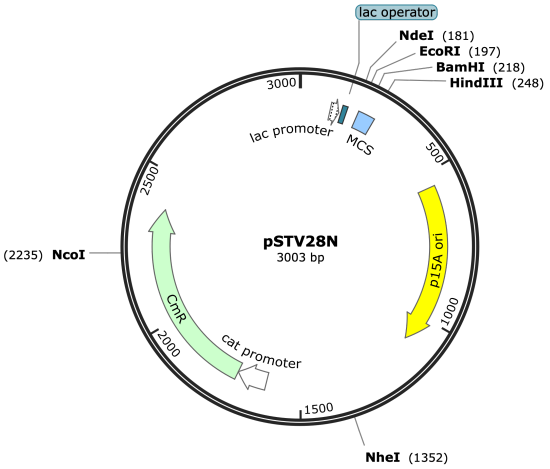

The DNA fragment amplified by PCR with pSTV28 as the template and a set of phosphorylated primers, SN1 and SN2, was self-ligated to construct pSTV28N.

Construction of BH4-regeneration related gene expression plasmid pSTV-BH4R

DNA fragment, which included PCD and DHPR genes optimized for the *E. coli* codon, was purchased from Integrated DNA Technologies, K.K. (Tokyo, Japan) and cloned into *Nde*I and *Hin*dIII site of pSTV28N vector.

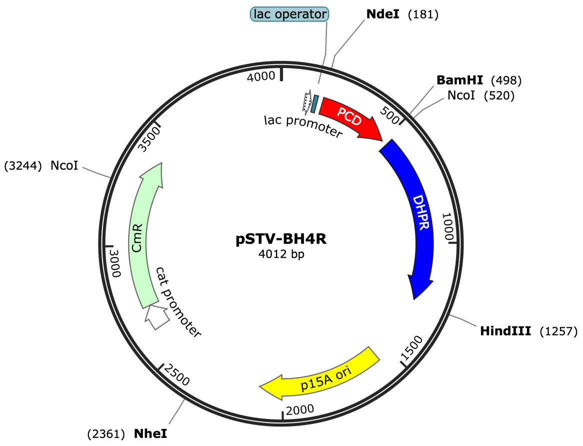

Construction of plasmids for PheH expression

A DNA fragment encoding the catalytic domain of the rat phenylalanine hydroxylase (PheH) gene was optimized for the *E. coli* codon (Integrated DNA Technologies) and cloned into *Nde*I and *Hin*dIII site of pQE1a-Red vector, designated as pQE1a-RatC.

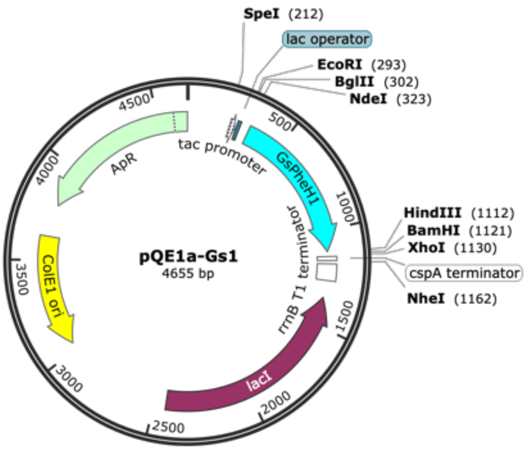

Bacterial PheH genes from *Bacillus* sp. INT005 (*BsPheH*, accession number LC739265), *Cupriavidus necator* (*CnPheH*, accession number WP_010811304), *Gulbenkiania* sp. SG4523 (*GsPheH1* and *GsPheH2,* accession numbers LC739263 and LC739264), *Xanthomonas oryzae* (*XoPheH*, accession number, WP_011257227), and *Pseudomonas putida* (*PpPheH*, accession number AAN70065) were obtained by PCR with their genomic DNA as templates and appropriate sets of primers (Table S2). Each of these genes were cloned into appropriate sites of pQE1a-Red vector.

To delete the internal *Eco*RI site in the *BsPheH* gene, a DNA fragment that would introduce a silent mutation at this site was obtained by overlap extension PCR and cloned into pQE1a-Red vector. As for *CnPheH* gene, a DNA fragment that introduced silent mutations at two internal *Eco*RI sites was obtained by assembling three amplicons prepared with appropriate sets of primers using In-Fusion cloning kit (Takara Bio Inc.). A DNA fragment of *XoPheH*, which introduced silent mutations at the internal *Eco*RI and *Bam*HI sites, was also obtained in a similar manner to *CnPheH* gene cloning.

To obtain the PheH gene of *Chromobacterium violaceum* (*CvPheH*, accession number AAD37774), a DNA fragment, codon-optimized for *E. coli*, was synthesized (Integrated DNA Technologies).

Construction of pQE1a-Gs1-BH4R for coexpression of *GsPheH1* and BH4-regeneration related genes

To coexpress *GsPheH1* and BH4-regeneration related genes in a single plasmid, a DNA fragment that included BH4-regeneration related genes was amplified by PCR using pSTV-BH4R as a template and SN3 and SN4 as primers, and then inserted into *Nhe*I site in pQE1a-Gs1, in the same direction as *GsPheH1*.

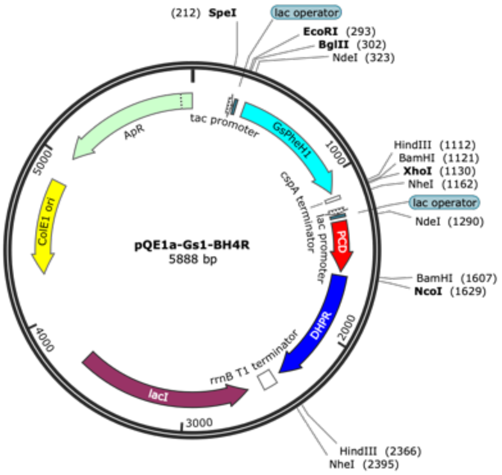

Construction of pCF1s-TYO-TDC and pCF1s-TDC-TYO plasmids

Plasmid pBbS1a-2 coexpresses TYO and TDC genes, in this order, as an operon and includes β-lactamase gene **[2]**. This plasmid was incompatible with pQE1a-Gs1-BH4R; therefore, their genes were cloned into *Eco*RI and *Xho*I sites of pCF1s-Red vector to obtain pCF1s-TYO-TDC.

To assess the gene order of TYO and TDC genes for tyrosol production, pCF1s-TDC-TYO, which coexpressed TDC and TYO genes as an operon in this order, was constructed based on the BglBricks cloning strategy **[1]**.

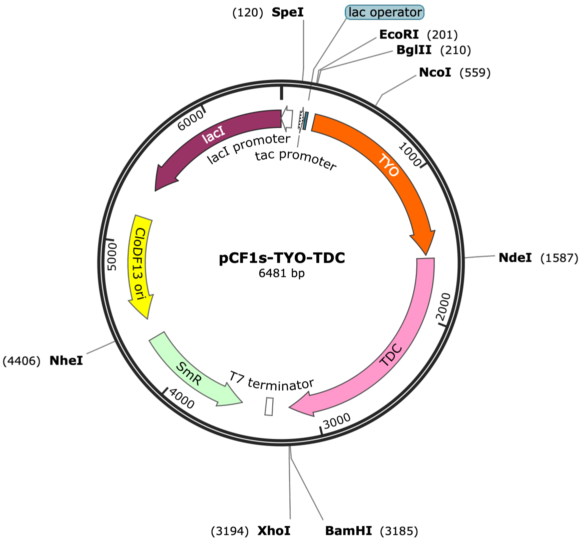

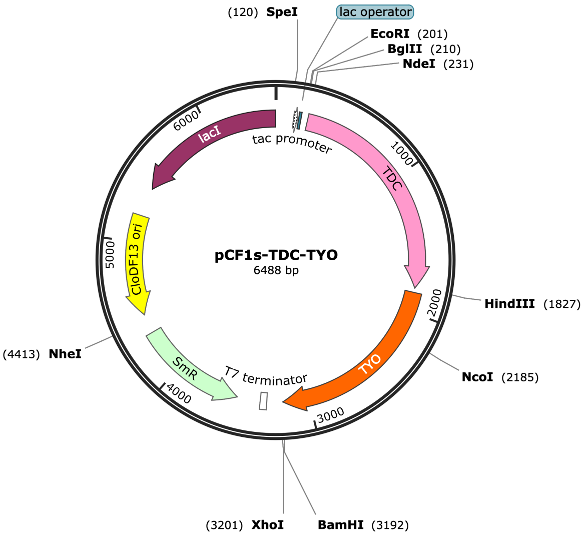

Construction of pCF1s-TDC-TYO-BH4R plasmids

For coexpression of TDC, TYO, and BH4-regeneration related genes, BH4-regeneration related genes were amplified by PCR using primers SN3/SN4 and pSTV-BH4R as a template and then inserted into *Nhe*I site in the pCF1s-TDC-TYO plasmid.

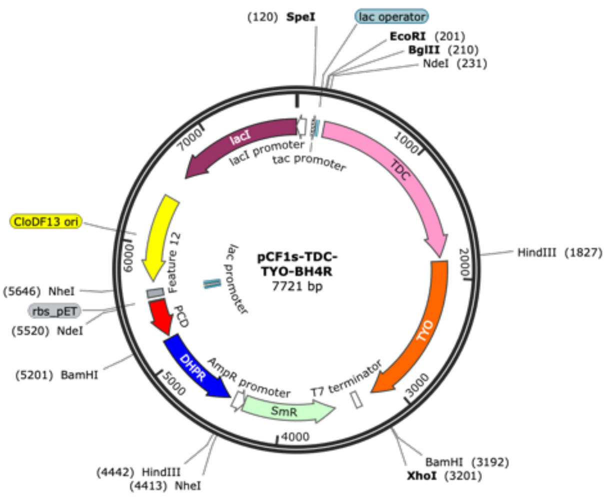

3. Construction of *E. coli* knockout mutants and *E. coli* strains integrated Tyr-producing module(s) on the chromosome

Escherichia coli knockout mutants were constructed by means of λRed-mediated recombination **[3, 4]**. To knockout *tyrA*, *aroD*, *cysE*, *serA*, or *feaB–tynA* genes, DNA fragments containing a Km-resistance marker flanked with flippase recognition target (FRT) sites and 50-bp homology arms were obtained by PCR with FRT-PGK-gb2-neo-FRT plasmid (Gene Bridges GmbH, Heidelberg, Germany) as a template and appropriate sets of primers (Table S2). The amplicons were used to transform appropriate *E. coli* mutants expressing λRed recombinase, and then Km-resistance strains were selected. The selection marker in the obtained strains were removed by FLP recombinase, if needed. Their gene knockout was confirmed by PCR, as shown below, and sequence analysis of the amplicons.

To express *GsPheH1* and BH4-regeneration related genes as a Tyr-producing module on the chromosome of *E. coli*, the cassette were integrated stepwisely. To obtain strain GsBR1, the module with 50-bp homology arms for *tyrA* locus was prepared by PCR with pQE1a-Gs1-BH4R as the template and primers tyrA-IN-up/tyrA-IN-dw (Table S2). A Tyr-auxotrophic strain Y0Km expressing λRed recombinase was transformed with the amplicon which was purified after treatment with the restriction enzyme *Dpn*I and then screened on M9 minimal medium plates. The gene integration into the chromosome was confirmed by PCR and sequence analysis of the amplicon.

Strain GsBR2, a derivative of strain GsBR1, had Tyr-producing module at Δ*feaB-tynA* region. A DNA fragment for chromosome integration was constructed using the overlap

extension PCR method. The Tyr-producing module, which was amplified with primers KmR-TyrMod-up/TyrMod-dw and pQE1a-Gs1-BH4R as the template, and the Km-resistance marker, which was amplified with primers KmR-up/KmR-dw and FRT-PGK-gb2-neo-FRT plasmid as the template, were assembled using overlap extension PCR with primers feaB-KO/IN-up and feaB-IN-dw. The amplicon was used for transformation of strain GsBR1 expressing λRed recombinase. After Km-resistance strains were selected, the marker in an obtained strain was removed by FLP recombinase.

Strain GsBR3 was constructed using *aroD* gene, an essential gene for *E. coli*, as a selection marker for chromosome integration of Tyr-producing module. The module, which was amplified with primers aroD-dw-TyrMod-up/aroD-IN-dw and pQE1a-Gs1-BH4R as the template, and *aroD*, which was amplified with primers aroD-IN-up/TyrMod-up-aroD-dw and a genomic DNA of *E. coli* BW25113 as a template, were assembled using overlap extension PCR with primers aroD-IN-up/aroD-IN-dw. The amplicon was integrated into the chromosome of strain GsBR2Δ*aroD*, which was an *aroD*-knockout mutant derived from strain GsBR2 as described above, expressing λRed recombinase. Finally, strain GsBR3 was obtained by screening with M9 minimal medium plates. The gene integration was confirmed using PCR as shown below, and sequence analysis of the amplicon.

Strains GsBR4 and GsBR5 were also obtained in a similar manner using *cysE* and *serA* genes as the selection markers, respectively. Primers used are shown in Table S2. Their gene integration was confirmed by PCR as shown below, and sequence analysis of the amplicon.

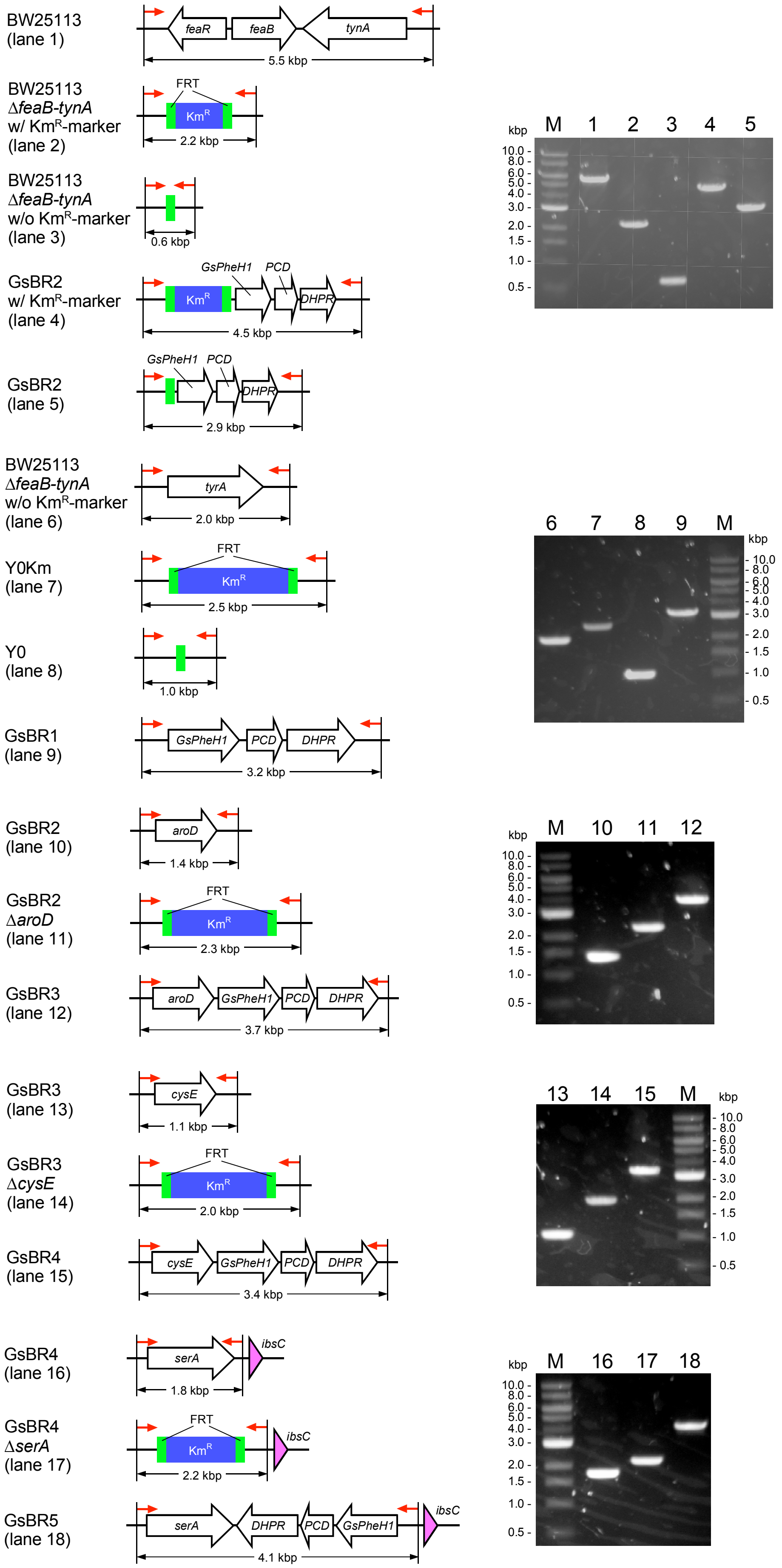
BW25113 (lane 1)
feaR
feaB
tynA
5.5 kbp
BW25113 ΔfeaB-tynA w/ KmR-marker (lane 2)
FRT
KmR
2.2 kbp
BW25113 ΔfeaB-tynA w/o KmR-marker (lane 3)
0.6 kbp
GsBR2 w/ KmR-marker (lane 4)
GsPheH1
PCD
DHPR
4.5 kbp
GsBR2 (lane 5)
2.9 kbp
BW25113 ΔfeaB-tynA w/o KmR-marker (lane 6)
tyrA
2.0 kbp
Y0Km (lane 7)
2.5 kbp
Y0 (lane 8)
1.0 kbp
GsBR1 (lane 9)
3.2 kbp
GsBR2 (lane 10)
aroD
1.4 kbp
GsBR2 ΔaroD (lane 11)
2.3 kbp
GsBR3 (lane 12)
3.7 kbp
GsBR3 (lane 13)
cysE
1.1 kbp
GsBR3 ΔcysE (lane 14)
2.0 kbp
GsBR4 (lane 15)
3.4 kbp
GsBR4 (lane 16)
serA
ibsC
1.8 kbp
GsBR4 ΔserA (lane 17)
2.2 kbp
GsBR5 (lane 18)
4.1 kbp
kbp
M
1 2 3 4 5
6 7 8 9
10 11 12
13 14 15
16 17 18
10.0 8.0 6.0 5.0 4.0 3.0 2.0 1.5 1.0 0.5

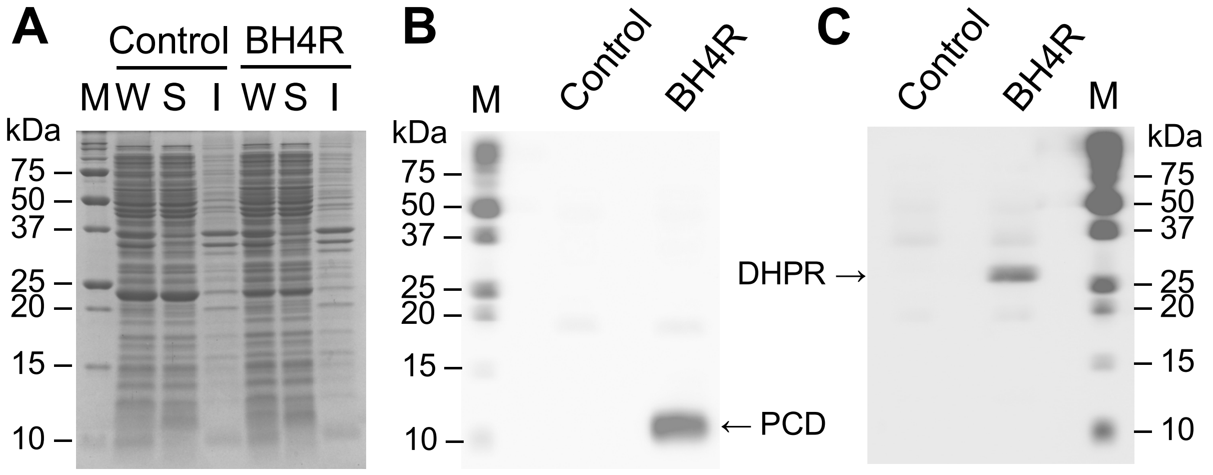

Figure S1. SDS-PAGE (A) and Western bolt (B and C) analyses of PCD and DHPR production.

(A) Whole cell (W), soluble (S), and insoluble (I) fractions prepared from strain Y0 harboring empty vector pSTV28N (Control) and pSTV-BH4R (BH4R), respectively, were analyzed by SDS-PAGE. Marked PCD (12.0 kDa) and DHPR (25.8 kDa) production was not confirmed on the gel. M shows a marker, and the apparent molecular weights of the bands are represented.

(B and C) PCD (**B**) and DHPR (**C**) in the soluble fractions used in (**A**) were detected with anti-PCD and anti-DHPR antibodies, respectively. Detected proteins are indicated by the arrows. M shows a marker.

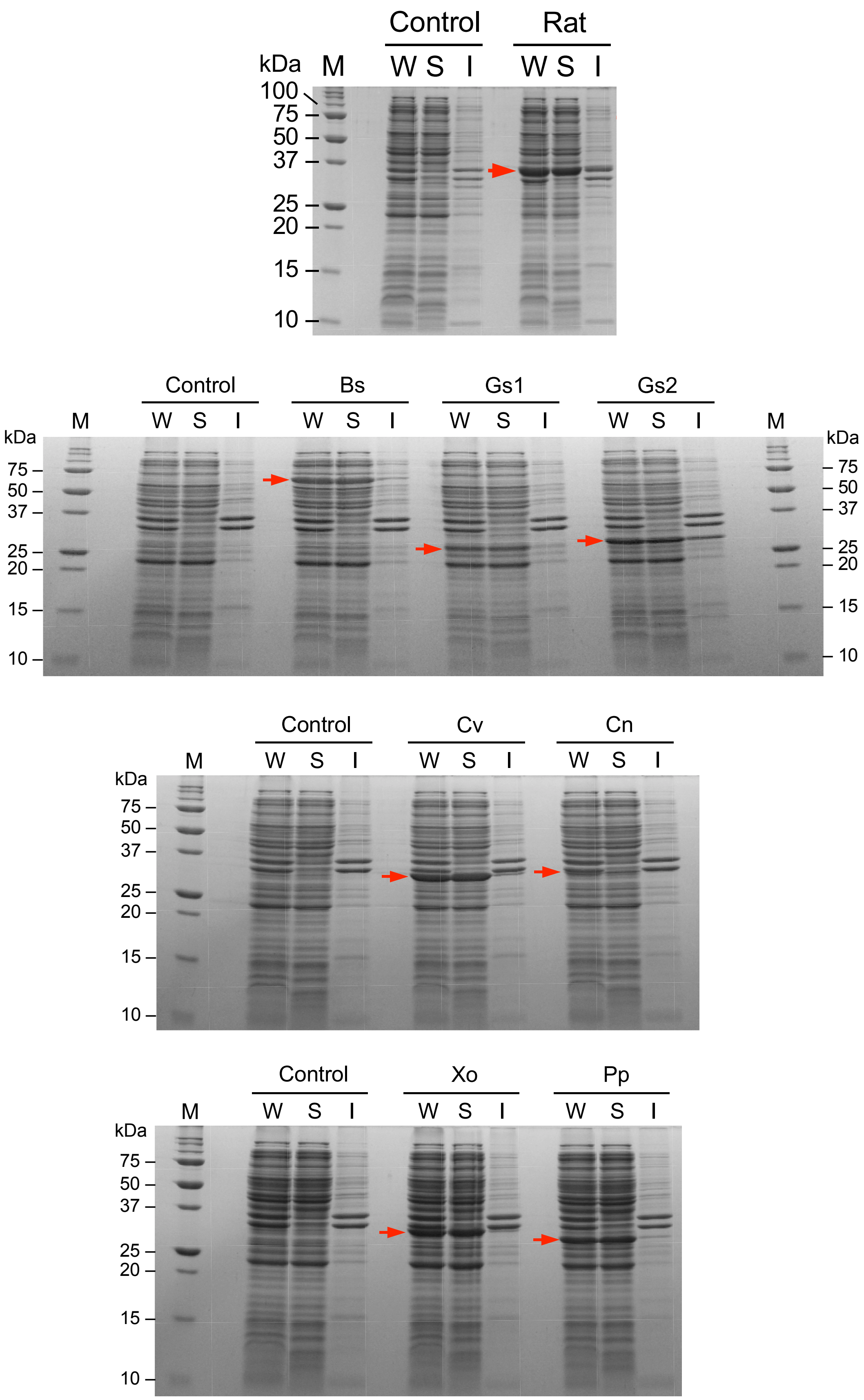

Figure S2. SDS-PAGE analysis of PheH production.

Whole cell (W), soluble (S), and insoluble (I) fractions of strain YBR harboring empty vector pQE1a (Control), pQE1a-RatC (Rat), pQE1a-Bs (Bs), pQE1a-Gs1 (Gs1), pQE1a-Gs2 (Gs2), pQE1a-Cv (Cv), pQE1a-Cn (Cn), pQE1a-Xo (Xo), or pQE1a-Pp (Pp), respectively, were analyzed by SDS-PAGE. M represents the marker, and the apparent molecular weights of the bands are indicated to the left of each gel. PheHs produced are indicated by the red arrows. RatPheHc, 38.6 kDa; BsPheH, 64.4 kDa; GsPheH1, 29.7 kD; GsPheH2, 32.3 kDa; CvPheH, 33.6 kDa; CnPheH, 34.9 kDa; XoPheH, 33.4 kDa; PpPheH, 30.1 kDa.

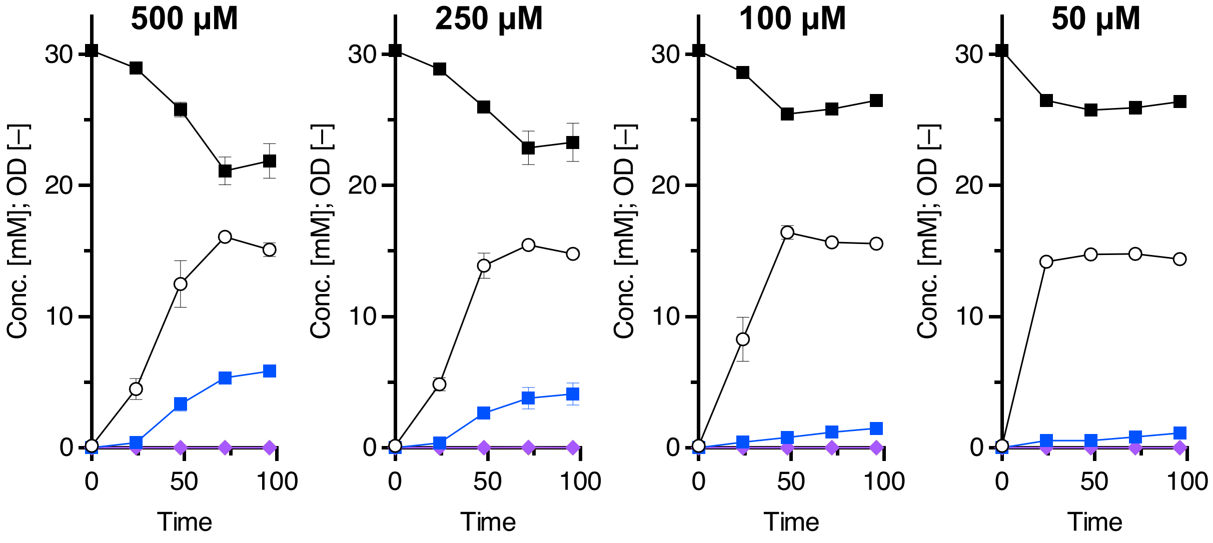

Figure S3. Fermentation profiles of strain GsBR5 transformed with pBbE1k-3 and pBbS1a-3.

The transformants were cultured up to 96 h at 30°C under the cultivation condition added IPTG at 50 to 500 µM. Phe, black squares; Tyr, blue squares; DOPA, purple diamonds; OD, open circles. Dopamine was not detected. Data are presented as mean values with standard deviations from three independent experiments. Symbols without an error bar indicate that they are larger than the size of the error bar.

Table S1. Identities (%) of amino acid sequences of PheHs.

	BsPheH	RatPheH	GsPheH1	PpPheH	XoPheH	GsPheH2	CvPheH	CnPheH
**BsPheH**	100.0	25.4	32.9	30.1	29.8	29.6	30.0	29.1
**RatPheH**	25.4	100.0	31.0	27.1	29.2	29.8	27.8	24.2
**GsPheH1**	32.9	31.0	100.0	41.6	41.9	43.4	42.9	42.3
**PpPheH**	30.1	27.1	41.6	100.0	41.7	43.5	43.5	43.4
**XoPheH**	29.8	29.2	41.9	41.7	100.0	50.0	59.2	57.1
**GsPheH2**	29.6	29.8	43.4	43.5	50.0	100.0	62.7	54.3
**CvPheH**	30.0	27.8	42.9	43.5	59.2	62.7	100.0	67.7
**CnPheH**	29.1	24.2	42.3	43.4	57.1	54.3	67.7	100.0

The amino acid sequences of PheHs were analyzed using Clustal Omega program (https://www.ebi.ac.uk/Tools/msa/clustalo/).

Table S2. Primers used in this study.

Primers used for plasmid construction.

Primers	Sequences (5' to 3')
QE1	TTTGTCGACGTGAAGACGAAAGGGCCTCGTGATACG
QE2	TGACTCGCTAGCTTGAGGCATCAAATAAAACGAAAGGCTC
QE3	GCGGATAACAATTTCACACAGGAAACAGAATTCAAAAGATCTAAGA AGGAGATATACATATGGACAACACCGAGGACGTCATCAAG
QE4	GATCCTTTAAGCTTTACTGGGAGCCGGAGTGGCGGGCCTC
QE5	TCACGTCGACACTAGTGAGCTGTTGACAATTAATCATCGGCTCGTAT AATGTGTGGAATTGTGAGCGGATAACAATTTCACACAGGAAACAG
QE6	CCTCAAGCTAGCAAATCCCCGCCAAATGGCAGGGATCTCTCGAGTT AGGATCCTTTAAGCTTTACTGGGAGCCG
CF1	ATACTAGTCCTAATGCAGGAGTCGCATAAGGGAGAG
CF2	CGACAAGCTTGCGGCCGCACTCGAGTCTG
SN1	CATATGTATATCTCCTTCTTGTGAAATTGTTATCCGCTCACAATTCC
SN2	ACCATGATTACGAATTCGAGCTCGGTACC
SN3	GGGCAGCTAGCGCAACGCAATTAATGTGAGTTAGCTCACTC
SN4	ATATAGCTAGCGTTGTAAAACGACGGCCAGTGCCAAG

Restriction enzyme recognition sites were shown as underlines.

Primers used for cloning of PheH genes.

Primers	Sequences (5' to 3')
Bs-F	ATAGAATTCAAAAGATCTAAGAAGGAGATATACATATGACAAAGAA AAGAGAAATTCCATCGCATTTAAAACC
Bs-Fe	CATGCGAACGAacTCGCCAACAGCATTAGCAATTCAC
Bs-Re	GTTGGCGAgtTCGTTCGCATGTAAATCAATTCGTCTG
Bs-R	GCGCTCGAGTTAGGATCCTTGAACTTCCTCTTCAGTTAATCGTAGCA TC
Cn-F	GAAGGAGATATACATATGTCCATCGCCACGGCCACCGAAG
Cn-Fe1	AGCGATGAATTtCTGCAGGGCCTGGCCAC
Cn-Fe2	GTACACGGTTGAATTtGGTCTGATCCGCACC
Cn-Re1	CAGaAATTCATCGCTGACACGGCCCTGCAG
Cn-Re2	AATTCAACCGTGTACCAGTACAGCCGCGACAG
Cn-R	AGGATCCTTTAAGCTTGATCAGATGTCTTCGGTATCGGCCCAGC
Gs1-F	GAGATATACATATGTATACCGCAGATGCGGCGCTCGTG
Gs1-R	TACAAGCTTAGATGGCCAGACCGTGGTGGCAGGTC
Gs2-F	GAGATATACATATGAACGACCGTAGCCCCTTTATCCCTC
Gs2-R	ATATAAGCTTAGGCCGCCACGTTGACGGAGCG
Xo-F	GAAGGAGATATACATATGAATACCGCCCCGCACCGCATCGAAAAC
Xo-Fb	TGGTGGATtCGCCGCCCCGATCAGATCGACTAC
Xo-Fe	GGAATTtGGCTTGATCGACACGCCACAG
Xo-Rb	GCGGCGaATCCACCACGTCACCGGGAAGCGCTTGTTG
Xo-Re	ATCAAGCCaAATTCCACCGTGTACCAGTACAGC
Xo-R	TCTCGAGTTAGGATCCAAAAAGCTTGGCGCGTTATTCCCGCAGTTG
Pp-F	GAAATTAACATATGAAACAGACGCAATACGTGGCAC
Pp-R	TATAAGCTTAGGCAGCGACCTTGGGTGGAAACTTCG

Restriction enzyme recognition sites are shown as underlines.

Small letters indicate silent mutations for deletion of appropriate restriction enzyme recognition sites.

Primers used for knockout and integration of target genes.

Primers	Sequences (5' to 3')
**For *tyrA* gene**	
tyrA-KO-up	CGGGCAGCTGACGGCTCGCGTGGCTTAAGAGGTTTATTATGGTT GCTGAATTAACCCTCACTAAAGGGCGGC
tyrA-KO-dw	CTGGATTATTACTGGCGATTGTCATTCGCCTGACGCAATAACAC GCGGCTAATACGACTCACTATAGGGCTCG
tyrA-IN-up	AGGATCTGAACGGGCAGCTGACGGCTCGCGTGGCTTAAGAGGT TTATTATCACGAGGCCCTTTCGTCTTCACGTCG
tyrA-IN-dw	CACTGGATTATTACTGGCGATTGTCATTCGCCTGACGCAATAAC ACGCGGATTTGTCCTACTCAGGAGAGCGTTCACC
**For *feaB–tynA* genes**	
feaB-KO/IN-up	TATCTGTTTTAACTAATTGGCGTTGCAGTACATGCAACGCCAAT TAGTTAATACGACTCACTATAGGGCTCG
feaB-KO-dw	ATAATGTGAACCTGACTAAACCGCCCACAGAGCGCGGTTGCTA ACAAGAATTAACCCTCACTAAAGGGCGGC
KmR-TyrMod-up	GCCGCCCTTTAGTGAGGGTTAATTCGTCTTCACGTCGACACTAG TGAGCTGTTGAC
TyrMod-dw	CTAGAGGGCGGATTTGTCCTACTCAG
feaB-IN-dw	GTACTGATAATGTGAACCTGACTAAACCGCCCACAGAGCGCGG TTGCTAATCTAGAGGGCGGATTTGTCCTACTCAGG
KmR-up	GAATTAACCCTCACTAAAGGGCGGC
KmR-dw	TAATACGACTCACTATAGGGCTCG
**For *aroD* gene**	
aroD-KO-up	TGGGGTTCGGTGCCTGACAGGCTGACCGCGTGCAGAAAGGGTA AAAAATGGAATTAACCCTCACTAAAGGGCGGC
aroD-KO-dw	GGAGGGTGTTCCCGCCGAAATATTATTGCTTATGCCTGGTGTAA AATAGTTAATACGACTCACTATAGGGCTCG

aroD-IN-up	TTCCCTCTGGAATATGTTAAACAGGTCATGG
TyrMod-up-aroD-dw	CGACGTGAAGACGAAATATTATTGCTTATGCCTGGTGTAAAATAGTTAATACCGTG
aroD-dw-TyrMod-up	GCATAAGCAATAATATTTCGTCTTCACGTCGACACTAGTGAGCTGTTGAC
aroD-IN-dw	ATTTTTAGTTCGGCGGGGAGGGTGTTCCCGCCGAAATATTATTGCTTATCATCGACTGCACGTAATCTAGAGG

**For *cysE* gene**	
cysE-KO-up	GGTCATTATCTCATCGTGTGGAGTAAGCAATGTCGTGTGAAGAACTGGAATTAACCCTCACTAAAGGGCGGC
cysE-KO-dw	GATCCCATCCCCATACTCAAATGTATGGTTAATACCGTTGAAATGCTGGTTAATACGACTCACTATAGGGCTCG
cysE-IN-up	GAACGGGCCGGTCATTATCTCATCGTGTG
TyrMod-up-cysE-dw	GTGTCGACGTGAAGACATTAGATCCCATCCCCATACTCAAATGTATG
cysE-dw-TyrMod-up	GGATGGGATCTAATGTCTTCACGTCGACACTAGTGAGCTGTTGAC
cysE-IN-dw	TGTAGGCCGGATAGATGATTACATCGCATCCGGCACGATCACAGGACATTTGTCCTACTCAGGAGAGCGTTCACCGAC

**For *serA* gene**	
serA-KO-up	CACAACATTTCAAAAGACAGGATTGGGTAAATGGCAAAGGTATCGCTGGAATTAACCCTCACTAAAGGGCGGC
serA-KO-dw	GCCCGTTGATTTTCAGAGAAGGGGAATTAGTACAGCAGACGGGCGCGAATAATACGACTCACTATAGGGCTCG
serA-IN-up	CGCACACAACATTTCAAAAGACAGGATTGG
TyrMod-dw-serA-dw	GACAAATCCGCCCTCTAGAATTAGTACAGCAGACGGGCGCGAATGG

serA-dw-TyrMod-dw	GTCTGCTGTACTAATTCTAGAGGGCGGATTTGTCCTACTCAGGAG
TyrMod-up	TCACGTCGACACTAGTGAGCTGTTGACAATTAATCATCG
serA-IN-dw	ACGGGCAAGTCAGTGACCTGCCCGTTGATTTTCAGAGAAGGGGAATTAGTCTTCACGTCGACACTAGTGAGCTGTTGAC

REFERENCES

1. Anderson JC, Dueber JE, Leguia M, Wu GC, Goler JA, Arkin AP, Keasling JD. BglBricks: a flexible standard for biological part assembly. J Biol Eng. 2010;4:1.
2. Satoh Y, Tajima K, Munekata M, Keasling JD, Lee TS. Engineering of a tyrosol-producing pathway, utilizing simple sugar and the central metabolic tyrosine, in Escherichia coli. J Agric Food Chem. 2012;60:979–84.
3. Datsenko KA, Wanner BL. One-step inactivation of chromosomal genes in Escherichia coli K-12 using PCR products. Proc Natl Acad Sci U S A. 2000;97:6640–5.
4. Sharan SK, Thomason LC, Kuznetsov SG, Court DL. Recombineering: a homologous recombination-based method of genetic engineering. Nat Protoc 2009:4;206–23.
